# Mechano-responsiveness of fibrillar adhesions on stiffness-gradient gels

**DOI:** 10.1242/jcs.242909

**Published:** 2020-06-22

**Authors:** Nuria Barber-Pérez, Maria Georgiadou, Camilo Guzmán, Aleksi Isomursu, Hellyeh Hamidi, Johanna Ivaska

**Affiliations:** 1 Turku Bioscience Centre, University of Turku and Åbo Akademi University, FIN-20520 Turku, Finland; 2 Department of Biochemistry, University of Turku, FIN-20520 Turku, Finland

**Keywords:** ECM elasticity, Fibrillar adhesion, Stiffness gradient hydrogels

## Abstract

Fibrillar adhesions are important structural and adhesive components in fibroblasts, and are required for fibronectin fibrillogenesis. While nascent and focal adhesions are known to respond to mechanical cues, the mechanoresponsive nature of fibrillar adhesions remains unclear. Here, we used ratiometric analysis of paired adhesion components to determine an appropriate fibrillar adhesion marker. We found that active α5β1-integrin exhibits the most definitive fibrillar adhesion localization compared to other proteins, such as tensin-1, reported to be in fibrillar adhesions. To elucidate the mechanoresponsiveness of fibrillar adhesions, we designed a cost-effective and reproducible technique to fabricate physiologically relevant stiffness gradients on thin polyacrylamide (PA) hydrogels, embedded with fluorescently labelled beads. We generated a correlation curve between bead density and hydrogel stiffness, thus enabling a readout of stiffness without the need for specialized knowhow, such as atomic force microscopy (AFM). We find that stiffness promotes growth of fibrillar adhesions in a tensin-1-dependent manner. Thus, the formation of these extracellular matrix-depositing structures is coupled to the mechanical parameters of the cell environment and may enable cells to fine-tune their matrix environment in response to changing physical conditions.

## Abstract (precis)

**Highlighted Article:** A simple method to generate stiffness gradient hydrogels that allows users to deduce gel stiffness, at any given point, using fluorescence microscopy and a mathematical equation.

## INTRODUCTION

It has been known for nearly two decades that cultured fibroblasts form distinct types of extracellular matrix (ECM) adhesions, the short-lived peripheral nascent adhesions, which are superseded by actin-tethered focal adhesions, and finally mature, centrally located, elongated fibrillar adhesions (Conway and Jacquemet, 2019; Katz et al., 2000; Zamir et al., 1999). Fibrillar adhesions mediate fibronectin remodelling and the formation of fibrils, which guide the deposition of other matrix components, such as collagens, fibrillin, fibulin and tenascin-C (Chung and Erickson, 1997; Dallas et al., 2005; Kadler et al., 2008; McDonald et al., 1982; Sabatier et al., 2009; Saunders and Schwarzbauer, 2019; Singh et al., 2010; Sottile and Hocking, 2002; Twal et al., 2001; Velling et al., 2002), and are thus important for the formation of the ECM. Fibrillar adhesions are partly defined by the presence of α5β1-integrin and tensin family proteins, and the absence of other integrin heterodimers (Pankov et al., 2000; Zamir et al., 2000). Ligand-bound α5β1-integrin translocates centripetally out of focal adhesions along the actin cytoskeleton, organizing bound fibronectin into fibrils (Pankov et al., 2000; Zamir et al., 2000). Active (i.e. fully primed or ligand occupied) α5β1-integrin is recognized by the SNAKA51 antibody and colocalizes with fibronectin in fibrillar adhesions (Clark et al., 2005).

The assembly and dynamics of nascent and focal adhesions, and thus cellular functions such as cell migration, spreading and differentiation, are known to be regulated by both chemical and mechanical cues (e.g. viscoelastic properties and tensile forces) emanating from the ECM (Choi et al., 2012; Hadden et al., 2017; Hetmanski et al., 2019; Holle et al., 2016; Lo et al., 2000; Martino et al., 2018; Pelham and Wang, 1998; Wang et al., 2012). Although, HIC-5 (also known as TGFB1I1), a paxillin family member, was recently shown to be required for the formation of tensin-1-positive fibrillar adhesions on rigid substrates (Goreczny et al., 2018), it still remains unclear whether fibrillar adhesions are also susceptible to changes in ECM elasticity.

Polyacrylamide (PA)-based hydrogels are the most commonly used *in vitro* cell culture platforms to study cellular behaviour in response to ECM elasticity, often referred to as stiffness or rigidity (Caliari and Burdick, 2016; Engler et al., 2006; Rowlands et al., 2008; Wen et al., 2014). These PA-hydrogels are usually generated with a uniform stiffness and, while being very informative for elucidating some of the molecular details regulating cell behaviour, are not representative of the *in vivo* situation. *In vivo*, the cellular microenvironment is extremely heterogeneous, not only in composition, but also in terms of stiffness (Young et al., 2016). Several different methods have been developed to generate stiffness gradient hydrogels that more closely mimic the mechanical heterogeneity observed *in vivo*, all with their own advantages and disadvantages (Chao et al., 2014; Hartman et al., 2016; Isenberg et al., 2009; Vincent et al., 2013). The main limitations include time-consuming, complex methodologies, or the need for specialized equipment not easily accessible in every laboratory. Moreover, in many stiffness gradient hydrogels it is not possible to know the exact stiffness to which the cells are exposed without the use of an atomic force microscope (AFM) (Hetmanski et al., 2019; Lo et al., 2000; Tse and Engler, 2010). A recent report described the fabrication of easy and robust stiffness gradient hydrogels to study human adipocyte-derived stem cell behaviour (Hadden et al., 2017). However, the resulting gels are relatively thick (∼1 mm) and thus are not suitable for high-resolution imaging. Another report correlated diffusion of fluorescein within a PA-hydrogel mix with hydrogel stiffness, removing the need for additional AFM analyses (Koser et al., 2016); however the setup of the makeshift chamber used in this study is time consuming and is not compatible with all microscopy setups and, as such, limits its application and reproducibility in other labs. Nevertheless, this study demonstrated the importance of mechanical signals for axon growth.

Here, we generate thin stiffness gradient hydrogels that can be easily fabricated in any laboratory, at low cost, on cell culture dishes without the need for specific equipment. These hydrogels contain fluorescently labelled beads, the density of which positively correlates with the stiffness of the gel. We generate an AFM-based correlation curve that allows researchers to assess the stiffness in every spot within the gradient of the gel simply by measuring the density of the beads using a confocal microscope. In parallel, we characterize the localization of an array of different adhesion proteins in fibroblasts and identify active α5β1-integrin as a more specific marker of fibrillar adhesions. Finally, by plating fibroblasts on physiologically relevant stiffness gradient hydrogels (0.5–22 kPa stiffness range) we find that fibrillar adhesion formation is mechanoresponsive, exhibiting a logarithmic and tensin-1-dependent growth in response to stiffness, rapidly increasing in length at the low stiffness regime (0.5–7 kPa), and gradually plateauing at higher stiffness (7–22 kPa).

## RESULTS

### Fabrication of bead-containing stiffness gradient hydrogels

**Fig. 1.**
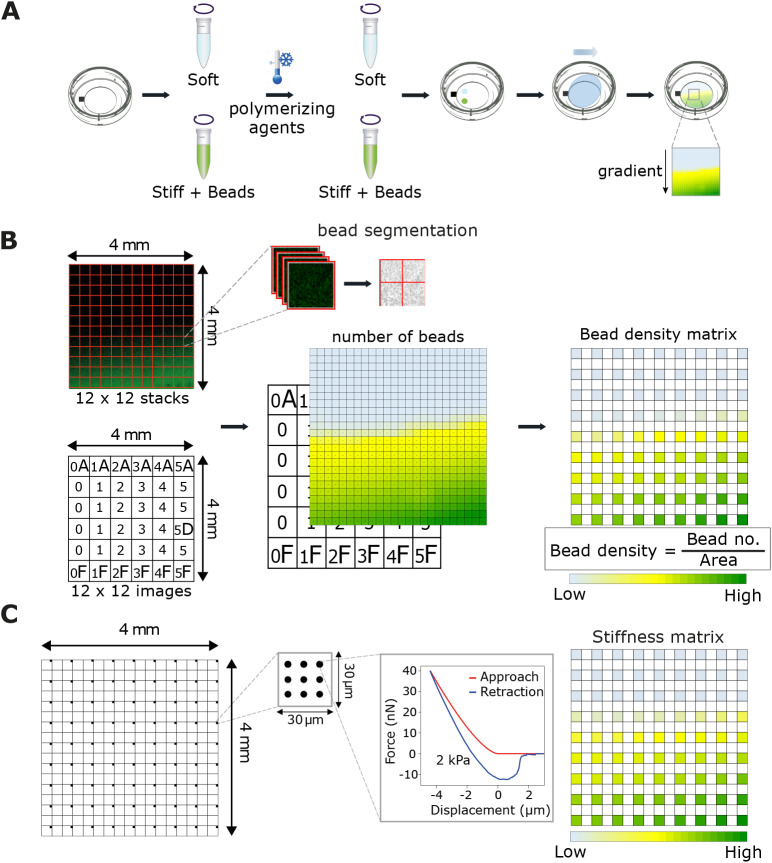
**Fabrication of PA gradient hydrogels and generation of the correlation curve between fluorescent beads and stiffness.** (A) Schematic for the fabrication of PA gradient hydrogels. A Petri dish with a gridded glass-bottom well was used to fabricate the hydrogels. Two PA solutions representing the extremes of the desired hydrogel gradient were dropped onto the glass, near a pre-drawn reference mark on the underside of the dish, and allowed to mix and diffuse on the surface, leading to the formation of a gradient. The stiff PA mix also contained fluorescent beads to infer hydrogel stiffness in later steps. (B) A 4 mm×4 mm region of bead gradient was selected and imaged using a spinning disk confocal microscope [12(*x*)×12(*y*)×7(*z*); total of 144 stacks]. Each stack was segmented and thresholded for bead fluorescence and a 2D matrix of bead density was created. In addition, a tile scan image of the gridded glass-bottom at the same area was acquired to be used as reference of position. (C) Using the image of the gridded glass-bottom, the same region of hydrogel was located and force measurements were performed using a JPK NanoWizard^®^ AFM system. Force measurements were carried out at different locations (0.5 mm apart in *x* and *y* coordinates when possible) within the region of interest (black squares; nine indentations distributed in 3×3 point grid) and the Young's elastic modulus for each force curve was calculated. A 2D matrix with spatial distribution of stiffness was then generated. The resulting matrices from B and C were used to calculate the best fit for the correlation curve between bead density and stiffness.

We aimed to overcome some of the limitations of currently available stiffness gradient methodologies by fabricating an easy to reproduce, low-cost and thin hydrogel suitable for high-resolution imaging. In addition, we sought a method that would allow the stiffness of the hydrogel to be measured at any given location without the need for AFM (Fig. 1). Towards this goal, we took elements from other approaches (Koser et al., 2016; Lo et al., 2000), and developed a modified method to generate stiffness gradient hydrogels. We prepared two acrylamide solutions corresponding to the softest and the stiffest parts of our desired hydrogel gradient and included fluorescently (505/515 nm; yellow-green) labelled beads (0.1 µm carboxylated FluoSpheres) within the stiff acrylamide solution. We then allowed the two mixtures to simultaneously diffuse and polymerise on a glass-bottom dish (Fig. 1A). Using this method, we consistently observed a region of bead gradient, which formed at the interface between the soft and stiff hydrogels, while other regions were either devoid of beads (corresponding to the softest hydrogel stiffness) or contained a homogenous distribution of beads (corresponding to the stiffest region of the hydrogel) (Fig. 1A,B; Fig. S1A–C). Owing to the nature of the method, individual hydrogels exhibit a degree of variability: for example, the gradient region might not be horizontal across the whole width of the gel (Fig. S1A). In some cases, the gel itself might be damaged or contain air bubbles, or the acrylamide diffusion results in an irregular or ‘jagged’ gradient; such gels should be discarded. We encourage users to check their gels with a fluorescence microscope before any experiments, and for example, realign them if a specific orientation of gradient is preferred.

Cells cultured on very thin (<20 μm) PA hydrogels may be able to ‘feel’ the underlying rigid glass or plastic, leading to confounded mechanosensing on such substrates (Buxboim et al., 2010). We measured the thickness of our stiffness gradient hydrogels along the length of the gradient and found it to be in the range of 100–150 µm (Fig. S1B), which is thick enough to prevent cells from being influenced by the glass but still amenable to high-resolution imaging.

### Generation of a correlation curve

We hypothesized that the concentration of beads in the hydrogel at any given point would correlate with the stiffness of the hydrogel, enabling: (1) rapid visual validation of the stiffness gradient with a fluorescence microscope and (2) a means to infer gel stiffness based on bead density rather than fluorescence intensity, which can be extremely variable, depending on microscope settings, and is subject to bleaching. To investigate this hypothesis, we set out to generate a correlation curve of AFM-defined stiffness versus bead density. In addition, since our protocol allows different stiffness gradients to be produced by simply changing the Young's modulus of the two starting PA solutions, we applied our analyses to two different gradients, one with a wide range (2–60 kPa) and one with a narrower, softer stiffness range (0.5–22 kPa).

**Fig. 2.**
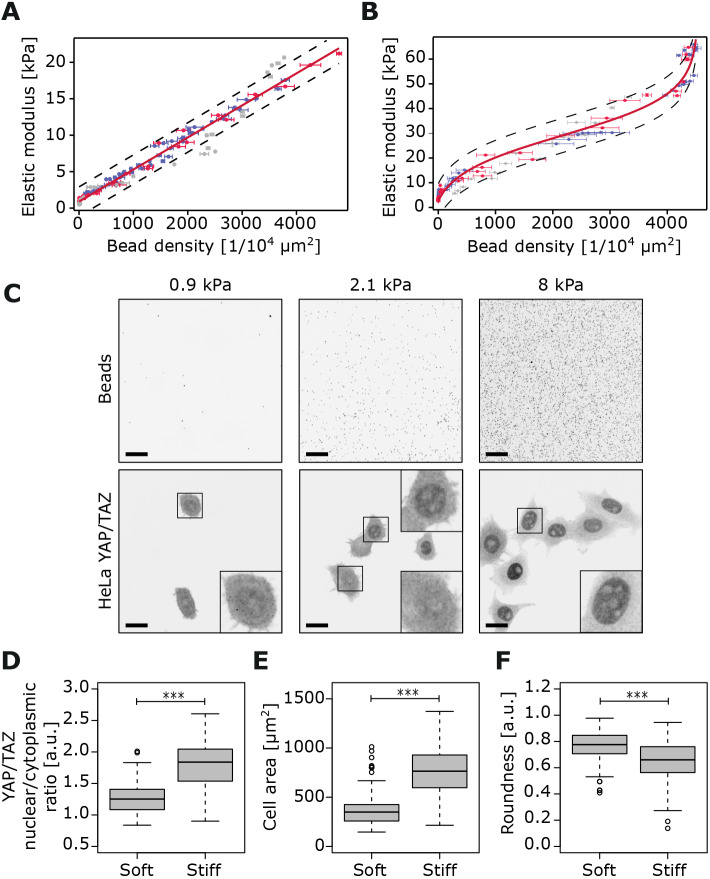
**Correlation curves between bead density and stiffness and validation of hydrogel gradient.** (A) Narrow range (0.5 kPa to 22 kPa) correlation curve. The best fit corresponds to a linear function (*n*=3 independent experiments, each experiment is colour-coded on the graph). (B) Wide range (2 kPa to 60 kPa) correlation curve. The best fit corresponds to a logit function (*n*=3 independent experiments, each experiment is colour-coded on the graph). For both A and B, each data point shows the mean±s.e.m. for averaged bead density (horizontal error bar; 2×2 grid of sub-images in the corresponding area; *n*=4) and averaged stiffness (vertical error bar; nine indentation points at each location; *n*=9). Dashed lines at either side of the curve correspond to the 95% confidence interval (c.i.). (C–F) HeLa cells were plated on the narrow range gradient hydrogels. Representative images of the beads (first row) and YAP/TAZ intracellular localization (second row) across the gradient of the hydrogel are shown. Bead density was used to calculate the hydrogel stiffness (linear function) (C). Tukey box plots displaying the median and the interquartile range (IQR: 25th–75th percentile) of YAP/TAZ nuclear to cytoplasmic ratio (D), cell area (µm^2^) (E) and cell roundness (F) located on the gradient's softer part (<1 kPa) compared with cells located on the stiffer part (>8 kPa). Whiskers extend to ±1.5× IQR and circles represent outliers (*n*=2 hydrogels; 80 cells per stiff and soft part for each hydrogel). ***P<0.001. Scale bars: 20 µm.

To pinpoint the same position within the hydrogel under two different imaging modalities, we prepared the hydrogels on gridded glass-bottom dishes (or used a reference mark), and then obtained a tile scan of bead distribution within the bead gradient using a spinning disk confocal microscope (Fig. 1B; see Materials and Methods), followed by AFM force measurements at defined points across the same area (Fig. 1C; Fig. S1C; see Materials and Methods). Our analyses demonstrated that, in both instances, AFM-defined stiffness indeed correlated with bead density (Fig. 2A,B). Moreover, the correlation curve for the narrower stiffness range (0.5–22 kPa) hydrogels could be best described as linear (Fig. 2A). In comparison, we found that the wide-range stiffness (2–60 kPa) correlation curve exhibited a more complex relationship between bead density and gel stiffness, best modelled as a logit curve (Fig. 2B). At the two extremes of the gradient, relatively small changes in bead density were accompanied by larger alterations in stiffness. Conversely, at intermediate bead densities, the relationship was more linear, and beads were a better overall predictor of substrate stiffness.

To validate the correlation between bead density and hydrogel stiffness, we compared the real gel stiffness, measured by AFM, to the calculated stiffness, based on bead density and the best-fit calibration curve, using additional wide-range stiffness gradient hydrogels. We found the AFM results highly consistent with bead-density-derived stiffness measurements; that is, AFM measurements were within the 95% confidence interval of the calibration curve (Fig. S1D).

### Biological validation of 2D-gradient hydrogels

Next, we sought to validate the biological applicability of our hydrogels by monitoring the subcellular localization of YAP1 and TAZ (also known as WWTR1), hereafter denoted YAP/TAZ, mechanosensitive transcriptional co-regulators, which are reported to undergo a cytoplasmic-to-nuclear switch in response to increasing ECM stiffness (Dupont et al., 2011; Elosegui-Artola et al., 2017). Indeed, on our narrow range softer hydrogels (0.5–22 kPa), we observed predominantly cytoplasmic YAP/TAZ localization at the softest region of the gel measured (0.9 kPa) that became progressively more nuclear as the stiffness gradient increased (Fig. 2C). The YAP/TAZ nuclear localization on the stiff portion of the gradient was also associated with increased cell spreading (enhanced cell area and decreased roundness) (Fig. 2D–F). These data indicate that stiffness gradient hydrogels could be used to monitor changes in cell morphology and to track the activation and/or subcellular localization of different mechanosensitive proteins in response to stiffness and perhaps help identify stiffness thresholds/responsiveness in other biological processes.

### Identification of a fibrillar adhesion marker

**Fig. 3.**
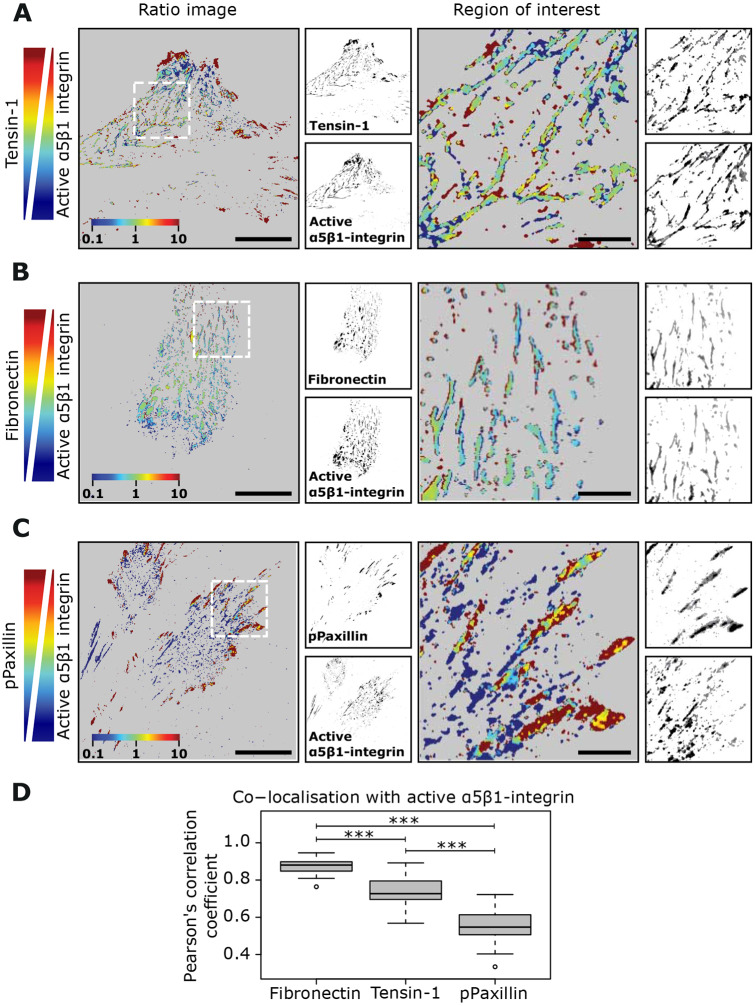
**Active α5β1 integrin as a fibrillar adhesion marker.** (A–D) TIF cells were plated on fibronectin-coated glass-bottom dishes overnight and stained for active α5β1-integrin and the indicated adhesion markers. Representative images and ratiometric analyses of colocalization between active α5β1-integrin (SNAKA51 antibody) and tensin-1 (A), active α5β1-integrin and fibronectin (B) and active α5β1-integrin and phosphorylated paxillin (pPaxillin) (C) and quantification of colocalization (Pearson's coefficient) are shown (D) (fibronectin *n*=28, tensin-1 *n*=21, pPaxillin *n*=24 cells). ***P<0.001 (one-way ANOVA and Tukey's honestly significant difference). Scale bars: 20 μm (main images; ROIs are 20 μm×20 μm). To obtain the Pearson's coefficient between each pair of images, the Fiji plugin JACoP was used. The Tukey box plots display the median and the interquartile range (IQR; 25th–75th percentile). Whiskers extend to ±1.5× IQR and circles represent outliers.

To be able to quantitatively investigate fibrillar adhesions in respect to substrate stiffness, we set out to first identify an ideal fibrillar adhesion marker. We allowed human telomerase-immortalized fibroblasts (TIFs) to form stable adhesions on fibronectin and then determined the localization of selected adhesion proteins, reported to be in focal or fibrillar adhesions, in a pairwise manner using a high-resolution OMX TIRF microscope and ratiometric fluorescence analysis (Zamir et al., 1999). We confirmed that tensin-1 and active α5β1-integrin (labelled with the SNAKA51 antibody, recognizing the primed conformation of the receptor in these fixed cells), previously reported to be enriched at fibrillar adhesions (Pankov et al., 2000; Zamir et al., 2000), demonstrate equal abundance in centrally located adhesions that are characteristic of fibrillar adhesions (Fig. 3A). These central adhesions, while rich in fibronectin (Fig. 3B; Fig. S2A), were largely devoid of the focal adhesion component vinculin (Fig. S2A). Active α5β1-integrin colocalized strongly with fibronectin (Fig. 3B), whereas tensin-1 was present in prominent vinculin-positive peripheral adhesions (Fig. S2B), in addition to central adhesions, suggesting a weaker colocalization between tensin-1 and fibronectin (dual labelling with fibronectin and tensin-1 antibodies was not possible due to antibodies being raised in the same species). Tensin-3, the other tensin isoform reported as being enriched in fibrillar adhesions (Clark et al., 2010), also overlapped with vinculin in peripheral adhesions (Fig. S2C).

Intrigued by the overlap between tensin-1 and vinculin, we analysed the distribution of tensin-1 in relation to other focal adhesion components. Dual labelling of tensin-1 with talin-1 confirmed that tensin-1 was co-expressed with talin-1 in focal adhesions (Fig. S3A). In contrast, fibronectin was absent from paxillin- and talin-1-positive adhesions (Fig. S3B). Taken together, our data demonstrates that tensin-1 is a component of both focal and fibrillar adhesions and thus may not be an ideal marker of fibrillar adhesions in stably adhered cells. However, active α5β1-integrin, which demonstrated a strong overlap with fibronectin in centrally located adhesions and is absent from peripheral adhesions, also showed limited colocalization with phosphorylated (p)-paxillin and p-FAK (FAK is also known as PTK2) (Fig. 3C,D; Fig. S3C). Thus, in line with fibrillar adhesions being viewed as phosphotyrosine-poor structures within the cell (Zamir et al., 2000), active α5β1-integrin appears to be a more appropriate fibrillar adhesion marker.

### Mechanoresponsive fibrillar adhesions

**Fig. 4.**
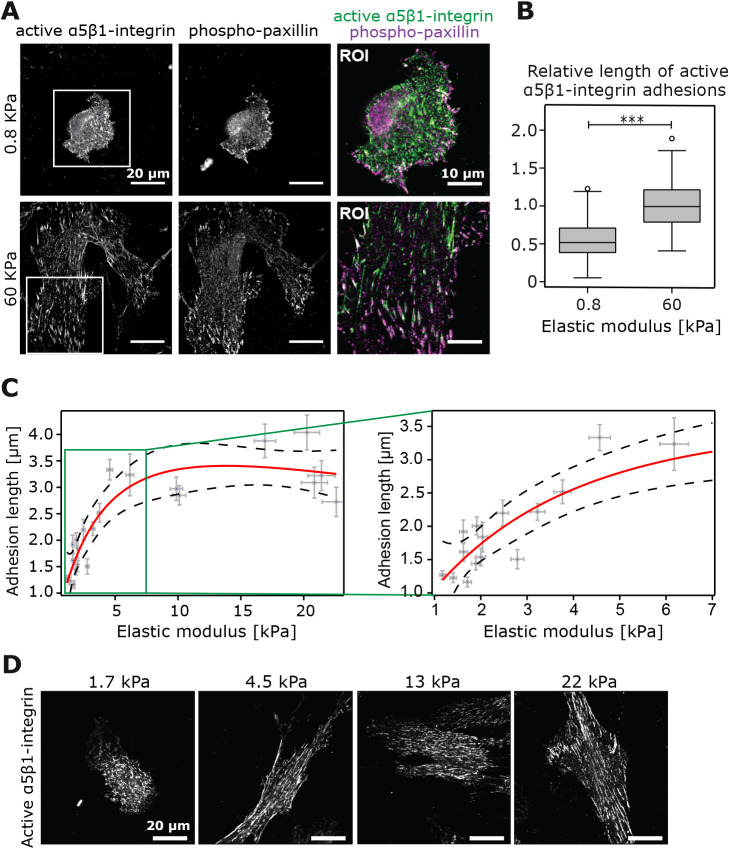
**Active α5β1 integrin adhesions respond to changes in stiffness.** (A) Representative images of TIFs plated on uniform stiffness hydrogels (0.8 kPa or 60 kPa) and stained for active α5β1-integrin and phospho-paxillin. Scale bars: 20 µm. (B) Tukey box plot of α5β1-integrin adhesion length (µm) from A [*n*=2 independent experiments, 9 cells (0.8 kPa) and 10 cells (60 kPa) analysed per experiment; 199 adhesions (0.8 kPa) and 211 adhesions (60 kPa)]. ***P<0.001. (C) Correlation analysis between substrate elastic modulus (kPa) and α5β1-integrin adhesion length (µm) in TIFs plated on a stiffness gradient hydrogel (0.5 kPa to 22 kPa) (*n*=1 independent experiment, 22 cells, >20 adhesions per cell). Error bars on the *y*-axis correspond to the s.d. on the adhesion length while error bars on the *x*-axis display error (95% c.i.) from the calibration curve used to convert from bead density into stiffness. (D) Representative images of TIFs stained for active α5β1-integrin from C across the stiffness gradient. Scale bars: 20 µm.

To address whether fibrillar adhesions respond to rigidity, we first plated TIFs overnight on fibronectin-coated hydrogels representing two extremes of substrate stiffness (0.8 kPa, very soft; and 60 kPa, very stiff). As shown previously (Yeung et al., 2005), we observed that TIFs spread more, exhibiting a flatter morphology, on the stiff versus the soft substrate (Fig. 4A). We measured the length of adhesions positive for active α5β1-integrin and negative for phosphotyrosine-paxillin and found that, on a soft substrate, fibroblasts had small, often dot-like adhesions, whereas on a stiff substrate the adhesions were primarily longer, resembling more-typical fibrillar adhesions (Fig. 4A,B). Next, we plated cells on the narrower stiffness gradient hydrogels (0.5–22 kPa) and monitored adhesion formation. We made the interesting observation that the length of active α5β1 integrin adhesions positively correlated with the stiffness of the substrate (Fig. 4C,D). This increase in adhesion length could be best described with a logarithmic distribution – a rapid increase at lower stiffness (1–7 kPa), followed by a more gradual increase and finally a plateau at higher stiffness (7–22 kPa) reaching a maximum average length of ∼3.5 µm in our system.

### Tensins support stiffness-induced adhesion elongation

**Fig. 5.**
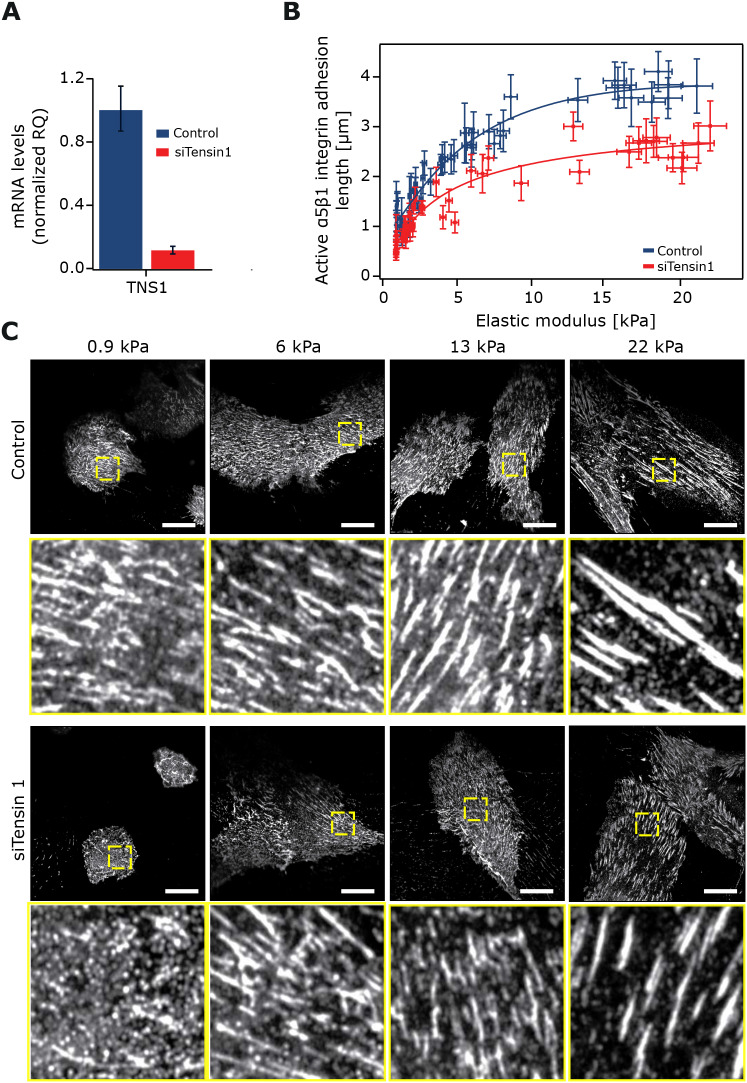
**Tensins are required for adhesion elongation in response to stiffness.** (A) Taqman qPCR analysis of tensin-1 silencing efficiency in TIF cells (*n*=4). Plots represent the normalized average value of RQ with its corresponding RQ min and RQ max. (B,C) TIF cells transfected with siRNA against tensin-1 (siTensin-1) and control-silenced TIFs were plated on stiffness gradient hydrogels and stained for active α5β1-integrin. (B) Comparison of active α5β1-integrin adhesion length across a hydrogel stiffness gradient between tensin-1-silenced TIFs (*n*=3 hydrogels) and controls (*n*=2); 20 cells were used per repeat with >20 adhesions per cell. Error bars on the *y*-axis correspond to the s.d. on the adhesion length while error bars on the *x*-axis display error (95% c.i.) from the calibration curve used to convert from bead density into stiffness. (C) Example images across the stiffness gradient are shown. Yellow squares denote magnified regions of interest of α5β1-integrin adhesions. Scale bars: 20 µm.

Tensins bind directly to the β1-integrin tail and stabilize integrins on the cell surface (Lo et al., 1994; Torgler et al., 2004). In addition, tensins maintain β1-integrin activity in fibrillar adhesions following initial talin-dependent switching of the receptor into an active conformer (Georgiadou et al., 2017). Moreover, a reduction in fibrillar adhesion number, as seen in tensin-depleted cells or upon AMPK activation, correlates with significantly reduced traction-forces on fibronectin (Georgiadou et al., 2017), indicating that fibrillar adhesions transduce forces to the ECM. To test whether tensins are required for the stiffness-dependent increase in active α5β1 integrin adhesion length, we silenced tensin-1 expression using siRNA oligonucleotides that we had previously validated for specificity with rescue experiments (Georgiadou et al., 2017). Interestingly, tensin-1 silencing, validated with qRT-PCR (Fig. 5A), clearly reduced active α5β1 integrin adhesion length in cells plated on the stiffness-gradient gels when compared to the control silenced cells (Fig. 5B,C). These data demonstrate that while tensins may not be restricted to fibrillar adhesions, they are important for active α5β1 integrin adhesion elongation on a range of matrix rigidities.

## DISCUSSION

Many fundamental cellular processes including proliferation, migration and differentiation are precisely regulated by cues emanating from a dynamic and heterogeneous microenvironment. These cues include fluctuations in the biochemical composition as well as the biophysical properties (viscoelasticity, topography and architecture) of the surrounding ECM.

Several methods have been developed to reduce the complexity of the microenvironment in order to imitate the effect of ECM stiffness on cell behaviour (Hadden et al., 2017; Koser et al., 2016; Lo et al., 2000; Tse and Engler, 2010; Vincent et al., 2013) and primarily involve the production of PA hydrogel-based stiffness gradients. While all of these techniques have their own unique advantages, limitations include production of thick hydrogels that are not compatible with high-resolution or live-cell imaging, the need for AFM verification of stiffness for individual experiments and the use of fine-tuned techniques for specific stiffness gradients that reduce reproducibility in other laboratories. Here, we set out to develop a simple and cost-effective method to produce stiffness gradients based on previous approaches (Koser et al., 2016; Lo et al., 2000). We demonstrate that our technique is flexible and can be applied to produce different rigidity gradients without the need for specialized equipment. The resulting hydrogels can be used as a reductionist model to image and dissect mechanosensitive pathways and signalling in cells. We show that, within a 0.5–22 kPa range, cell spreading increases and YAP/TAZ localization becomes progressively nuclear with increasing stiffness. While these results are expected, these proof-of-concept data indicate that our microscopy-amenable hydrogels could potentially be used to simultaneously chart the effect of substrate stiffness heterogeneity on two or more proteins within the same dish or to track dynamic changes in individual cells when they encounter different mechanical cues. Whether precise stiffness thresholds, for example, for inducing complete YAP/TAZ nuclear translocation, could be determined for different cell lines, remains to be investigated but would be fundamental in our understanding of mechanosignalling in development and disease.

We also used our stiffness gradient hydrogels to explore whether fibrillar adhesions, the main sites of fibronectin fibrillogenesis, respond to changes in ECM rigidity. We demonstrate that in TIFs, fibrillar adhesion length, identified by active α5β1-integrin staining, increases rapidly up until ∼7 kPa. After this point, adhesion lengthening decelerates and eventually becomes relatively stable, suggesting that the formation of fibrillar adhesions is indeed mechanosensitive. Importantly, we find this mechanosensitive adhesion lengthening to be tensin-dependent. Recently, tensins have been implicated in supporting integrin activity and traction forces in fibroblasts *in vitro* (Georgiadou et al., 2017), in addition to integrin activity *in vivo* in the myotendinous junctions of *Drosophila* flight muscles (Green et al., 2018). The exact nature of how fibrillar adhesions retain their connection to the actin cytoskeleton, perhaps through integrin–tensin interaction, remains to be investigated. However, our data show that these structures respond to gradual changes in ECM rigidity.

In our set-up, we opted to use bead density rather than fluorescence intensity as a readout of hydrogel stiffness. We believe that the change from using fluorescein intensity (Koser et al., 2016) to the analysis of bead density (our method) gives a more flexible and viable approach, as fluorescence intensity can be a highly variable read-out depending on bleaching rate and on the imaging modality used. We show that while there is a linear correlation between bead density and hydrogel stiffness at narrower stiffness gradients (0.5–22 kPa), at wider stiffness gradients, a logit fit appears to be a more accurate representation of the relationship (2–60 kPa). It is unclear whether this non-linear relationship results from partially different diffusion kinetics between acrylamide and the fluorescent marker beads, a phenomenon that is then exacerbated by the increased acrylamide concentration in the wide-range gradients. To our knowledge, no diffusion-based polyacrylamide stiffness gradients with elastic moduli reaching up to 60 kPa have been reported before, for example, when fluorescein was used as a means to measure hydrogel stiffness (range of 0.1–10 kPa; Koser et al., 2016). This observation is therefore important to keep in mind for any future modifications of the technique.

Fibronectin structure and function undergo mechano-regulated alterations (Craig et al., 2001; Smith et al., 2007) that could, for example, influence fibronectin-dependent assembly of other ECM components, such as collagen (McDonald et al., 1982; Saunders and Schwarzbauer, 2019; Velling et al., 2002). However, the notion that, through mechanosensitive fibrillar adhesions, fibronectin remodelling may also be subject to regulation by substrate rigidity has received less attention. The stiffness-dependent lengthening of fibrillar adhesions observed here has potentially important implications in tissue fibrosis (Chen et al., 2014; Pelouch et al., 1993), cancer (Cox and Erler, 2011) and drug resistance. In the context of cancer, this process may impinge on fibronectin-guided invasion of cancer cells in the tumour microenvironment (Oudin et al., 2016), or on nutrient sensing through the modulation of α5β1-integrin endocytosis and recycling (Georgiadou and Ivaska, 2017; Rainero et al., 2015).

## MATERIALS AND METHODS

### Fabrication of PA gradient hydrogels

Glass-bottom dishes (0.13–0.16 mm thickness, 14 mm diameter, Cellvis, D35-14-1-N) were treated for 20 min at room temperature (RT) with 200 µl of Bind-silane solution – a mixture of 714 µl 3-(trimethoxysilyl)propyl methacrylate (3-TMP, Sigma-Aldrich, M6514) and 714 µl of acetic acid in 10 ml of 96% ethanol. This mix was used to covalently attach PA hydrogels to the glass surface and to prevent hydrogel detachment. After the Bind-silane was aspirated, the glass surface was washed twice with ethanol and left to dry completely. A reference mark was also manually drawn on the underside of the dish with a permanent marker (Fig. 1A).

Two acrylamide solutions, one soft (0.5 kPa or 2 kPa) and one stiff (22 kPa or 60 kPa), were prepared to create rigidity gradients of ∼0.5–22 kPa and ∼2–60 kPa. The desired Young's modulus (*E*) of the pre-mixes was adjusted by mixing pre-defined ratios of 40% (w/v) acrylamide monomer (Sigma-Aldrich, A4058) and 2% (w/v) N,N-methyl-bis-acrylamide cross-linker (Sigma-Aldrich, M1533) in PBS (Table S1). The solutions were kept on ice to avoid fast polymerization in later steps. A standard volume (1.7 µl, 3.6×10^10^ beads/µl) of fluorescently labelled (505/515 nm) beads (0.1 µm carboxylated FluoSpheres; Thermo Fisher Scientific, F8803) was sonicated (3 min) and added into the stiff pre-mix. Both acrylamide solutions, soft and stiff, were vortexed briefly and then used immediately in the next step.

Polymerization of the soft pre-mix was started by addition of 5 µl 10% ammonium persulfate (APS; Bio-Rad) and 1 µl N,N,N′,N′- tetramethylethylenediamine (TEMED; Sigma-Aldrich T-9281) to the solution. The polymerizing soft mixture was quickly vortexed, and a 7.8 µl droplet of the solution was pipetted on top of the glass-bottom well ∼3 mm across and 1 mm above the reference mark. The same polymerization procedure was repeated with the stiff pre-mix, and a 7.8 µl droplet of the solution was placed ∼2 mm below the soft acrylamide droplet. A circular coverslip (13 mm) was then placed on top of the droplets by gently dropping it from the edge of the reference mark towards the opposite side of the glass well, leading to *in situ* mixing of PA gels and diffusion across the dish.

The hydrogel was left to polymerize for 1 h at RT. Upon polymerization the gel was covered with PBS for 5 min before the coverslip was carefully removed with a bent needle. Finally, the hydrogel was washed with PBS to remove any remaining unpolymerized acrylamide, and then immersed in PBS and stored at 4°C until needed.

### Generation of stiffness gradient correlation curves from PA hydrogels loaded with fluorescent beads

Correlation curves were generated for a wide stiffness range hydrogel (2–60 kPa) and a narrow stiffness range hydrogel (0.5–22 kPa). For this purpose, hydrogels were prepared on gridded glass-bottom dishes (Cellvis, D35-14-1.5GO) as above to allow the same area to be located under different microscopes (spinning disk confocal and AFM).

#### Analysis of bead number

The bead gradient within the hydrogel was pinpointed using a spinning disk confocal microscope (3i CSU-W1) equipped with a 40× objective (C-Apochromat 40×/1.1 NA; Zeiss) and a sCMOS (Hamamatsu Orca Flash 4; Hamamatsu Photonics) camera. A tile scan [12(*x*)×12(*y*)×7(*z*) images] covering an area of 4 mm×4 mm was acquired (488 nm laser line, intensity: 800 W/cm^2^; GFP 510–540 nm emission filter). The *z*-upper-limit for each stack was set 1 µm underneath the surface of the gel resulting in 144 stacks of 324.48 µm×324.48 µm×10 µm in size. The focal plane of the microscope was then changed to focus on the gridded glass-bottom, and a tile scan of bright-field images [12(*x*)×12(*y*)] covering the same region as the beads was acquired (Fig. 1B).

A semi-automatic Fiji macro with custom script (available from GitHub; https://github.com/Ivaska-Lab-UTU/StiffnessGradientHydrogels) was then used to process the acquired images. Briefly, for each stack a maximum intensity projection was produced and then segmented, with the appropriate threshold, into a 2×2 grid (total 576 images from the original 144 stacks), allowing a more accurate quantification of the beads within the same image. A custom Python script (available from GitHub; https://github.com/Ivaska-Lab-UTU/StiffnessGradientHydrogels) was then used to calculate the density of beads per area unit (1/10^4 ^µm^2^) and to create a 2D matrix displaying the spatial distribution of bead density (Fig. 1B).

Alternatively, in order to display a whole stiffness gradient hydrogel in lower resolution (Fig. S1A), a 9 (*x*)×9 (*y*)×20 (*z*) tile scan was acquired using a Nikon Eclipse Ti2-E widefield fluorescence microscope, 10× Nikon CFI Plan-Fluor/0.3 NA objective and sCMOS Orca Flash4.0 camera with 2×2 binning (Hamamatsu Photonics). The system was controlled using NIS-Elements AR 5.11.00 software (Nikon).

#### AFM analysis of hydrogel stiffness gradient

The elastic modulus of the hydrogel (Young's modulus, *E*) was assessed using a JPK NanoWizard with a CellHesion module mounted on a Carl Zeiss confocal microscope, Zeiss LSM510 (AFM; JPK instruments) and silicon nitride cantilevers (spring constant: 1 Nm^−1^, spherical 10 µm diameter tip; Novascan Technologies). The cantilever spring constant and deflection sensitivity were calibrated in fluid via the thermal noise method (Hutter and Bechhoefer, 1993). Prior to distance force measurements, a CCD camera mounted on the AFM was used to visualize the grid of the culture dish and to locate the same 4 mm×4 mm region of interest previously imaged with the spinning disk microscope. Force measurements were then carried out at different locations (0.5 mm apart in *x* and *y* coordinates) within the region of interest. In each location, nine indentations distributed in a 3×3 point grid (30 µm×30 µm) were performed. The elastic modulus for each force curve was calculated using JPK data processing software (JPK DP version 4.2) assuming a Hertz model of impact (Fig. 1C).

A custom Python script was then used to consolidate all measurements of elastic moduli from multiple files into a single file, to calculate the mean between the nine stiffness values obtained per location and to create a 2D matrix displaying the spatial distribution of stiffness (Fig. 1C).

#### Correlation between bead density and AFM-defined hydrogel elastic moduli

To assess the correlation between bead density and hydrogel elasticity, the tile scan of the grid was overlaid with the bead density matrix. By doing this, it was possible to identify the bead location corresponding to the point where the elasticity measurements were taken. The Igor Pro software (IgorPro 6.37, Wavemetrics) was then used to plot bead density against elasticity, and to calculate the best fitting curve for the data. In both cases – for wide range (2 to 60 kPa) and narrow range (0.5 to 22 kPa) gradients – data from three independent hydrogels was processed as previously described and combined to generate the two final correlation curves.

The best fit for the narrow range correlation curve (0.5–22 kPa) corresponded to the following linear function:
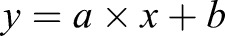

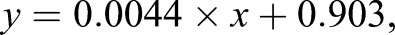
where *y* corresponds to the elastic modulus, *x* to bead density (number of beads in an area of 100 µm×100 µm), and the fitted constants *a* and *b* to the slope and the intercept respectively.

The best fit for the wide range correlation curve (2–60 kPa) corresponded to the following Logit function:
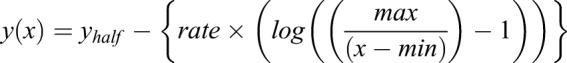

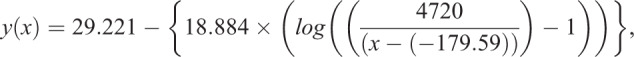
where *y* corresponds to the elastic modulus, *x* to bead density (number of beads in an area of 100 µm×100 µm), *y*_*half*_ (fitted constant; 29.221±4.67×10^−15^) is the half point of the logit where there is a change in curvature, *rate* (fitted constant; 18.884±6.19×10^−15^) is the rate at which the elastic modulus is increasing and *min* and *max* (fitted constants; 4720±0.00 and −179.59±1.66×10^−13^, respectively) correspond to the limit conditions in the abscissa. These equations were used in ensuing experiments to calculate hydrogel elasticity.

### Hydrogel activation and functionalization

For functionalization, 0.2 mg/ml Sulfo-SANPAH (Thermo Fisher Scientific, 22589) and 2 mg/ml N-(3-dimethylaminopropyl)-N′-ethylcarbodiimide hydrochloride (EDC) (Sigma-Aldrich, 03450) in 50 mM HEPES were added on top of the hydrogels and incubated for 30 min at RT, protected from light, and with gentle agitation. Gels were then placed into a UV-chamber for 10 min to activate the Sulfo-SANPAH and finally washed three times with PBS prior to overnight coating with the indicated ECM molecule/s at 4°C.

### Cell culture

Human cervical adenocarcinoma (HeLa) cells were obtained from the ATCC and maintained in high-glucose Dulbecco's modified Eagle's medium (DMEM) supplemented with 10% fetal bovine serum (FBS), 1% non-essential amino acids, 1% 2 mM L-glutamine and 1% penicillin-streptomycin (Pen-Strep). All cells were routinely tested for mycoplasma contamination. Human telomerase-immortalized fibroblasts (TIFs) were a gift from Jim Norman (Beatson Institute, Glasgow, UK) and were cultured in DMEM 4500 supplemented with 20% FBS, 2 mM L-glutamine and 20 mM Hepes buffer (Sigma-Aldrich).

### siRNA transfections

siRNA silencing was performed using 20 nM siRNA oligonucleotides and Lipofectamine^®^ RNAiMAX Reagent (ThermoFisher Scientific) according to manufacturer's protocol and cells were cultured for 3 days before the experiments. The siRNA against human tensin-1 (L-009976, Smartpool) and the control siRNA (D-001810-10, non-targeting Pool) were ON-TARGETplus siRNAs from Dharmacon, Thermo Fisher Scientific.

### Quantitative real-time PCR

Total cellular RNA was extracted using the NucleoSpin RNA kit (MACHEREY-NAGEL). The extracted RNA (1 µg) was used as a template for cDNA synthesis by the high-capacity cDNA reverse transcription kit (Applied Biosystems) according to the manufacturer’s protocol. Human tensin-1 expression levels were determined in control- and tensin-1-silenced cells by TaqMan quantitative real-time PCR using the RT-PCR HT7900 system (Applied Biosystems). The expression level of GAPDH was used as a reference. The TaqMan Universal Master Mix II included all the necessary components for quantitative RT-PCR reactions. The human TNS1 primers were purchased from Sigma-Aldrich (forward, 5′-CCAGACACCCACCTGACTTAG-3′, and reverse, 5′-CAGCTCATGGTTGGATGGA-3′; Universal ProbeLibrary probe 82). Relative tensin-1 expression was calculated by the 2ΔΔCT method using the expression level of GAPDH as a reference for the quantification.

### Antibodies, compounds and reagents

The following antibodies were used: anti-YAP/TAZ (sc-101199, Santa Cruz Biotechnology), anti-tensin-1 (SAB4200283, Sigma-Aldrich), anti-fibronectin (F3648, Sigma-Aldrich), anti-vinculin (clone hVIN-1, V9131), anti-talin (clone 8d4, T3287, Sigma-Aldrich), anti-paxillin (612405, BD Biosciences), anti-phosphotyrosine (Y118)-paxillin (2541, Cell Signaling Technology), anti-phosphotyrosine (Y397)-FAK (clone D20B1, 8556, Cell Signaling Technology) and anti-tensin-3 (ABT29, Millipore). Anti-human active α5-integrin (SNAKA51) was a gift from Martin Humphries, University of Manchester, UK (Clark et al., 2005). Phalloidin Atto 647N (65906) was obtained from Sigma-Aldrich. Alexa Fluor^®^-conjugated secondary antibodies (Alexa Fluor 488, 555, 568, 647-conjugated anti-mouse-, rabbit- and rat-IgG antibodies, Thermo Fisher Scientific) were used in immunofluorescence. Bovine plasma fibronectin (FN; 341631) was purchased from Merck, Millipore and collagen type I (from calf skin) from Sigma-Aldrich (C8919).

### YAP/TAZ immunofluorescence

HeLa cells were seeded (4×10^3^ cells) for 24 h on gradient hydrogels pre-coated (overnight, 4°C) with 2.5 µg/ml fibronectin+2.5 µg/ml collagen I. Cells were fixed with a final concentration of warm 4% PFA added straight into the media at RT. Blocking and permeabilization was performed with 0.3% Triton-X in 10% horse serum for 15 min. After washing with PBS, cells were incubated overnight at 4°C with the indicated primary antibody, mouse anti-YAP/TAZ (1:100), in 10% horse serum. Cells were then washed with PBS and incubated with Alexa Fluor 568-conjugated secondary antibody (1:200; 1.5 h at RT), Phalloidin Atto 647N (1:200) and DAPI (1:1000). Finally, cells were washed and kept in PBS until imaging.

HeLa cells were imaged using a spinning disk confocal microscope (3i CSU-W1), a 40× objective (C-Apochromat 40×/1.1 NA; Zeiss) and a sCMOS (Hamamatsu Orca Flash 4; Hamamatsu Photonics) camera. A semi-automatic custom macro script in ImageJ (Fiji) was used to determine YAP/TAZ nuclear to cytoplasmic intensity ratio. Briefly, maximum intensity projections were created and the nucleus (defined by DAPI staining) and cytoplasm (region corresponding to a 1 µm ring around the nucleus, excluding DAPI staining) were segmented by drawing one line around the DAPI staining (nucleus) and another line 1 µm away from DAPI staining. YAP/TAZ mean intensities were then calculated in the different regions. The cell area was calculated from maximum intensity projections of actin staining in ImageJ. Hydrogel stiffness was determined as described above using bead density and the linear equation [*y*=0.0044 *x* + 0.903].

### Ratiometric analysis of adhesions pairs in TIFs

TIF cells were seeded overnight on glass-bottom dishes (MatTek Corporation) pre-coated with 10 µg/ml fibronectin (overnight at 4°C), fixed and permeabilized with 4% PFA and 0.2% Triton-X for 10 min, blocked with 1 M glycine for 30 min, washed and then incubated with the indicated primary antibodies for another 45 min. Following further washes, cells were incubated with Alexa-conjugated secondary antibodies (6 μg/ml), Phalloidin Atto 647N (1:200) and 0.5 μg/ml DAPI in PBS for 30 min. Finally, cells were washed with PBS and Milli-Q water and imaged using a DeltaVision OMX v4 microscope with a Ring-TIRF module (GE Healthcare), 60× Olympus APO N TIRF/NA 1.49 oil objective (RI 1.518 immersion) and front illuminated pco.edge sCMOS camera (PCO AG) (0.08 μm pixel size, 95 MHz).

Ratiometric analysis was performed using a modified version of a previously described protocol (Zamir et al., 1999). In short, two-colour images of TIFs stained with the proteins of interest were first processed to remove background and noise. Using the ‘subtract background’ and the ‘threshold’ functions of ImageJ software (NIH) a mask was created setting to zero all pixels below threshold and maintain the values of pixels above threshold. For accuracy, each of the labelled channels was processed separately. Ratio images were then calculated using the open source software R (R Core Team) by dividing, pixel by pixel, the values of the first channel by the second channel. Given that there exist multiple pixels with a zero value in both channels/labels, we defined a multiple case scenario to calculate the ratio image: (1) a resulting value of zero was assigned whenever the pixel in both channels/labels was zero; (2) a value of 0.1 was assigned whenever the ratio between the pixel in label A (numerator) and the pixel in label B (denominator) was ≤0.1; (3) a value of 10 was assigned whenever the ratio between the pixel in label A (numerator) and the pixel in label B (denominator) was ≥10, or in the case the numerator was >0 and the denominator was zero; and (4) in all the remaining cases the pixel was assigned the ratio value between the numerator and the denominator pixel. After all ratio values were calculated and assigned, the images were displayed in log scale using a colour look-up table (Jet2 for all pixels >0 and grey for pixel values of 0); such representation allows the presentation of ratio value variations over two orders of magnitude (from 0.1 to 10).

### Analysis of adhesion length

TIF cells were plated overnight on hydrogels (0.8 kPa, 60 kPa or 0.5–20 kPa stiffness gradient hydrogels) precoated with 10 µg/ml fibronectin and stained for active α5β1-integrin (SNAKA51 antibody) as described above. The laser scanning confocal microscope (CLSM, Zeiss LSM 880 AiryScan) with LD LCI Plan-apochromat 40×/1.2 (NA) objective and super-resolution AiryScan detector was used to image fibrillar adhesions in cells at different locations across the stiffness gradient. Adhesion length was then manually measured in Fiji by using the freehand measuring tool. The mean adhesion length and standard deviation was calculated for each cell.

### Statistical analysis

Unless otherwise stated, a Student's *t-*test (two-tailed, equal variances) was used for statistical analysis.

## Supplementary Material

Supplementary information

Reviewer comments
