# Supplementary material for: Mechano-responsiveness of fibrillar adhesions on stiffness-gradient gels
Source: J Cell Sci. 2020 Jun 22;133(12):jcs242909. doi: 10.1242/jcs.242909 (PMC7328166; doi:10.1242/jcs.242909)
Supplement: Supplementary information [file joces-133-242909-s1.pdf]

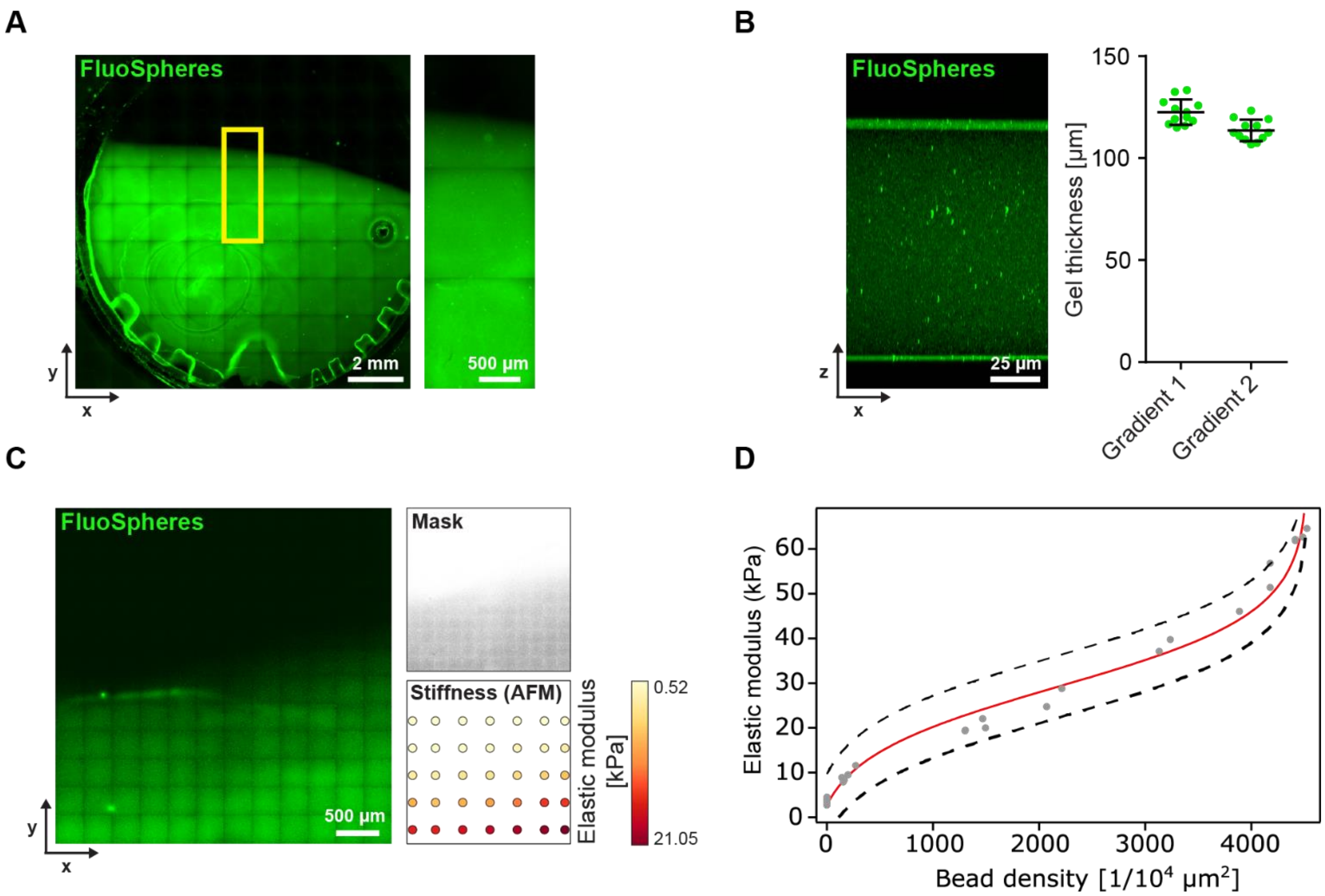

Figure S1

A) (Left) Widefield fluorescence microscopy montage depicting a typical narrow stiffness range gradient hydrogel. Fluorescent microbeads act as a surrogate marker for acrylamide diffusion and resulting substrate stiffness. (Right) Close-up of the gradient region indicated by the yellow square on the left. **B)** Confocal microscopy image (left) and quantification (right) showing gel thickness in two (325 x 975) μm^2 regions on two different gradient hydrogels. Maximum intensity projection. **C)** Confocal microscopy montage (left) depicting one of the narrow stiffness range gradients with a corresponding thresholded mask of the beads (top right) and AFM measurements (bottom right). Each colored circle represents the mean elastic modulus in the (20 x 20) μm^2 area indicated by the center of the circle. **D)** AFM validation of calibration curve-derived stiffness values. AFM was used to measure hydrogel stiffness at different regions within the stiffness gradient hydrogel and was compared to the values derived from the calibration curve. Red line represents the calibration curve, grey circles are the AFM measurements at the indicated bead density and the dashed lines at either side of the curve correspond to the 95% confidence interval of the original fit.

Journal of Cell Science • Supplementary information

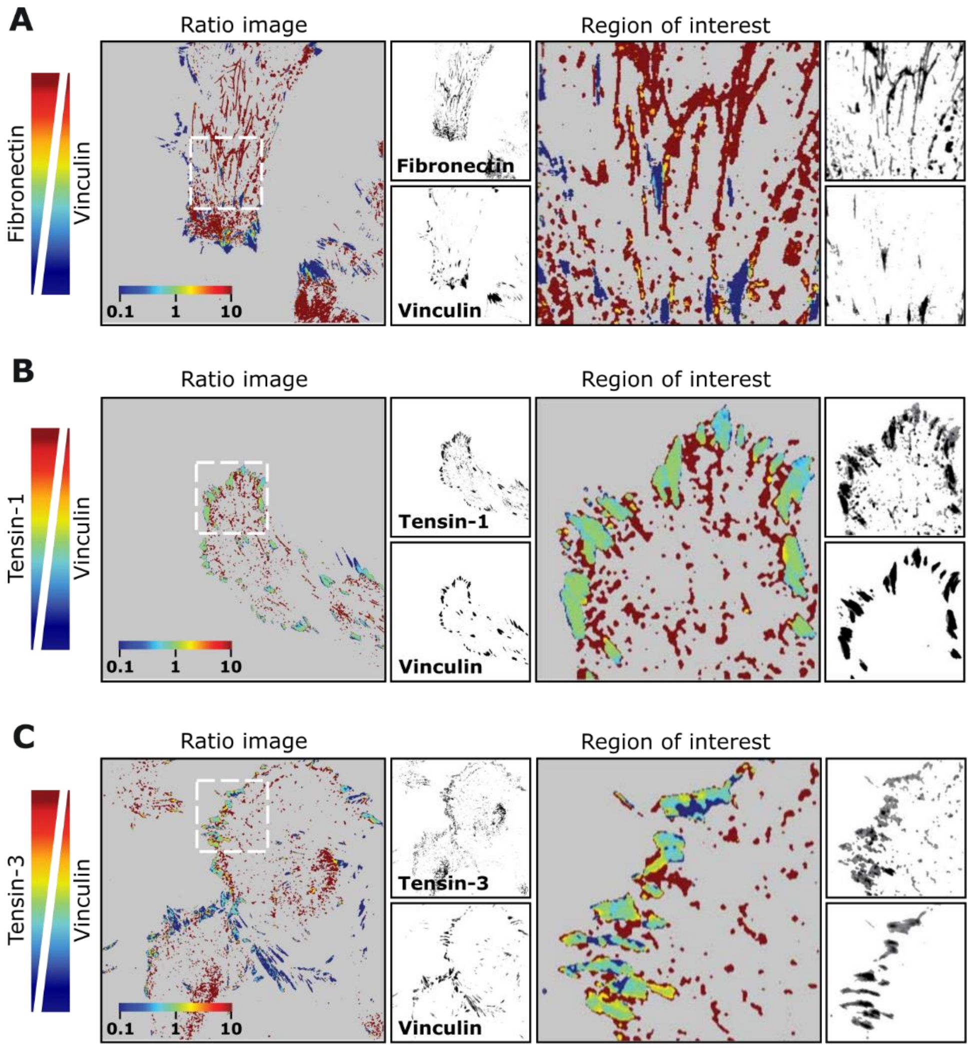

Figure S2.

(A – C) TIF cells were plated on fibronectin-coated glass-bottom dishes overnight and stained for the indicated adhesion markers. Representative images and ratiometric analyses of colocalization between vinculin and fibronectin (A), vinculin and tensin-1 (B) or tensin-3 (C) are shown.

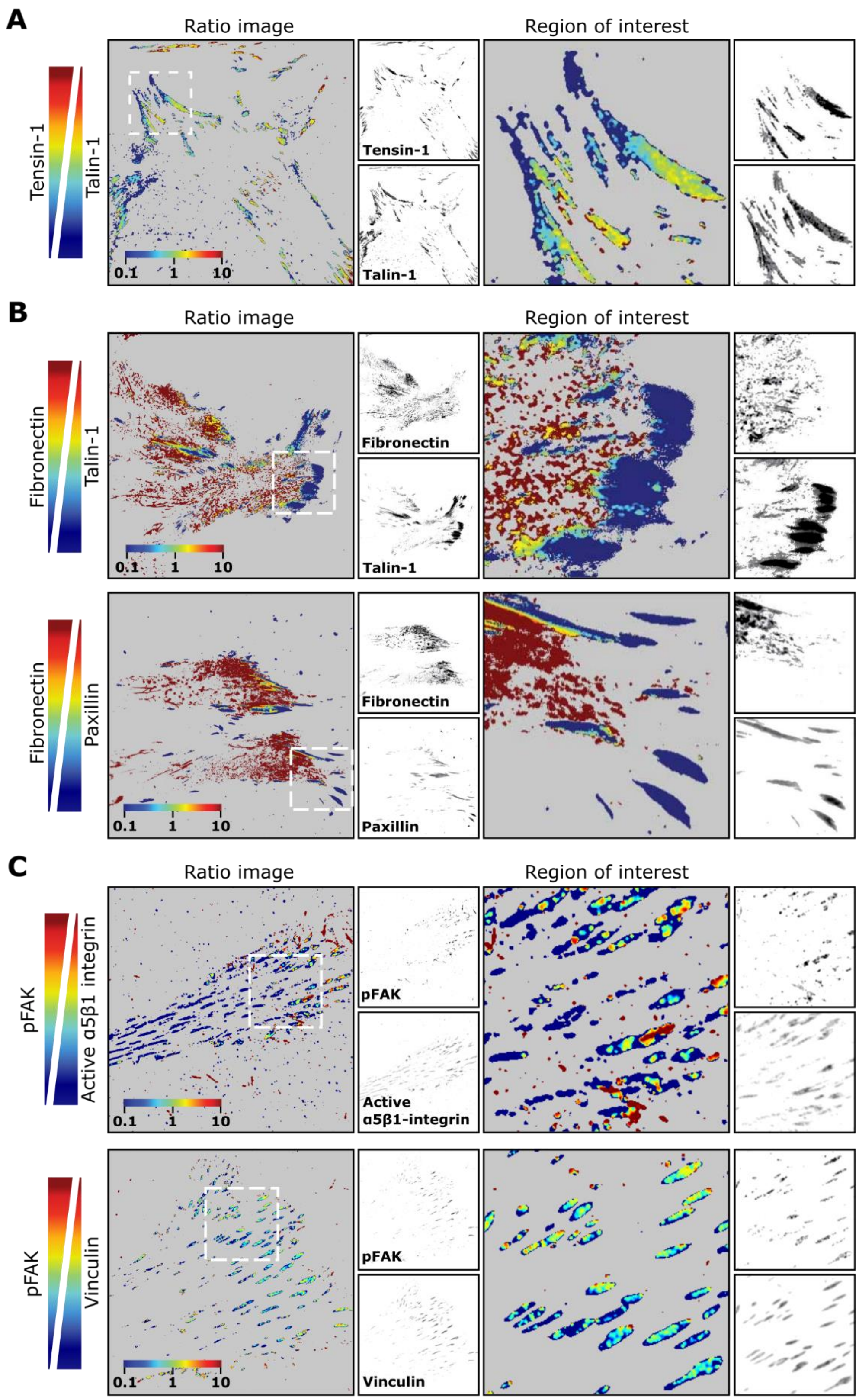

Figure S3.

(A – C) TIF cells were plated on fibronectin-coated glass-bottom dishes overnight and stained for the indicated adhesion markers. Representative images and ratiometric analyses of colocalization between tensin-1 and talin-1 (A), between fibronectin and talin-1 or paxillin (B) and between pFAK and active α5β1 integrin or vinculin (C) are shown.

Journal of Cell Science • Supplementary information

Table S1. Relative acrylamide and bis-acrylamide concentrations for the fabrication of uniform (constant *E)* hydrogels and expected Young's modulus after polymerization [a]

Final acrylamide %	Final Bis-acrylamide %	Stock acrylamide (40%) (µl)	Stock Bis-acrylamide (2%) (µl)	PBS (µl)	*Young's modulus* *E* ± σ (kPa)
5	0.04	63	10	397	0.5 ± 0.4
5	0.07	63	17.5	365	1.8 ± 2
12	0.2	150	50	300	21 ± 1
18	0.4	225	100	175	60 ± 2

[a] The values for the Young´s modulus provided in this table were obtained using AFM indentations on 2-3 hydrogels per condition and at least 100 indentations per sample.
