# Supplementary material for: Mechano-responsiveness of fibrillar adhesions on stiffness-gradient gels
Source: J Cell Sci. 2020 Jun 22;133(12):jcs242909. doi: 10.1242/jcs.242909 (PMC7328166; doi:10.1242/jcs.242909)
Supplement: Reviewer comments [file joces-133-242909_review_history.pdf]

Mechano-responsiveness of fibrillar adhesions on stiffness-gradient gels

Nuria Barber-Pérez, Maria Georgiadou, Camilo Guzmán, Aleksi Isomursu, Hellyeh Hamidi and Johanna Ivaska
DOI: 10.1242/jcs.242909

Editor: Michael Way

Review timeline

Original submission:	13 December 2019
Editorial decision:	16 January 2020
First revision received:	20 April 2020
Accepted:	22 April 2020

Original submission

First decision letter

MS ID#: JOCES/2019/242909

MS TITLE: Mechano-responsiveness of fibrillar adhesions on stiffness-gradient gels

AUTHORS: Nuria Barber-Perez, Maria Georgiadou, Camilo Guzman, Aleksi Isomursu, Hellyeh Hamidi, and Johanna Ivaska
ARTICLE TYPE: Tools and Resources

I hope you had a great holiday and the good news for 2020 is we have now reached a decision on the above manuscript.

To see the reviewers' reports and a copy of this decision letter, please go to: https://submit-jcs.biologists.org and click on the 'Manuscripts with Decisions' queue in the Author Area. (Corresponding author only has access to reviews.)

As you will see, all three reviewers are positive although reviewer 2 does question the 'novelty' aspect. The reviewers suggest that a revised version might prove acceptable, if you can address their concerns including toning down the novelty aspect. If you think that you can deal satisfactorily with the criticisms on revision, I would be pleased to see a revised manuscript. We would then return it to the reviewers.

Please ensure that you clearly highlight all changes made in the revised manuscript. Please avoid using 'Tracked changes' in Word files as these are lost in PDF conversion.

I should be grateful if you would also provide a point-by-point response detailing how you have dealt with the points raised by the reviewers in the 'Response to Reviewers' box. Please attend to all of the reviewers' comments. If you do not agree with any of their criticisms or suggestions please explain clearly why this is so.

© 2020. Published by The Company of Biologists under the terms of the Creative Commons Attribution License (https://creativecommons.org/licenses/by/4.0/).

Reviewer 1

Advance summary and potential significance to field

This manuscript describes a creative approach to producing stiffness gradients in very thin polyacrylamide gels that can permit high-resolution imaging. A novel feature is that the predicted stiffness can be determined using embedded micro beads, potentially avoiding the need to perform AFM to calibrate stiffness. The authors use this system to show that (1) a better marker for fibrillar adhesions associated with fibronectin assembly is the SNAKA51 antibody of Clark et al. than the more widely distributed tensin-1, and (2) the formation of fibrillar adhesions is mechanosensitive in the TIF immortalized cell line. Consequently this study provides a new method for creating stiffness gradients usable for high-magnification microscopy, identifies a good fibrillar adhesion marker, and shows mechanosensitivity of fibrillar adhesion formation.

Comments for the author

This very well-presented Tools and Resources article provides a cleaver new method for forming very thin polyacrylamide gels with stiffness gradients confirms and extends information in the previous literature that a particular antibody is a good fibrillar adhesion marker rather than tensin-1, and is the first to show that the formation of fibrillar adhesions is mechanosensitive.
Although the latter finding is not surprising given the need for force in organizing fibronectin fibrils, it is novel and useful to establish. The authors of this solid paper should consider the following points in a resubmitted manuscript so that publication can be recommended.

1. A key advance is to provide a new methodology for cell biology labs without ready access to AFM to create stiffness gradients amenable to low working distance microscopy. Several points need clarification:
a) The authors seem to imply that this methodology can be used by other labs using this paper's standard curves to assign Young's moduli without using AFM.
If that is the intent, it should be made explicit. If so, this reviewer and readers would prefer to see a little more characterization. Since 3 experiments were apparently combined for standard curves, it would be useful to see the raw data for each experiment as supplemental data. That is, the field will want to see the variability in both the data points of bead concentration vs. stiffness as well as the overall nature of the gradients (e.g., just show the presumably existing images of the fields showing the distribution of beads, probably using stitched-together images), since the simple methods of blending two dots of different polyacrylamide solution is clever but would likely give varying results from experiment to experiment depending on the spacing of the dots and the way in which the coverslip is dropped. In fact, Figure 1 shows a gradient that is diagonally skewed as would be predicted to occur periodically when fusing droplets under a coverslip; the extent of variability would be good to see.
Should users discard some gels that do not look even? In addition, the Figure 2C "Beads" image at 4 kPa shows a density of beads at the bottom similar to the uniform distribution at 8 kPa, suggesting variability. These issues may not be a major problem, but it will be important to see the actual data for users to understand what to expect in terms of variability.
b) This reviewer is quite surprised at the tight fitting of the AFM data in supplemental figure 1 to the predicted curve at the very low and high stiffness zones, especially considering the comment on lines 143-144 stating that "significant changes in stiffness were not accompanied by changes in bead density.". But if values in these regions are reliable, this method could be used for a wide range of stiffnesses, but is that not the case?
c) Most importantly, it is not clear to this reviewer how there can be such non-linear relationships between bead concentration and AFM-measured stiffness, since the bead concentration should be a simple proxy for polyacrylamide concentration.
This problem ideally needs some explanation beyond the very unclear comment about large differences in stiffness of the starting gels (lines 144-145) , since such non-linearity seems to imply that it may not be safe to use the authors' standard curves for other than narrow stiffness ranges.

2. For this methodology to be useful to other labs, the three custom scripts for the processing approaches will need to be readily available. Will they be available from a public database with instructions for use? Without such methods and a reliable pair of standard curves, this methodology will not be sufficiently useful to the field.

© 2020. Published by The Company of Biologists under the terms of the Creative Commons Attribution License (https://creativecommons.org/licenses/by/4.0/).

3. How thick are the polyacrylamide gels (beyond just less than 100 um)? That the thickness can matter is shown by the Buxboim-Discher paper PMID: 20454525, in which proximity to the substrate can affect what cells can sense. Some reassurance that this issue will never be a problem would be helpful.

4. The use of statistics for Figure 3D was likely incorrect because there were three sets of data compared rather than a single pair. The authors should either use the Bonferroni correction for t-tests or ANOVA with an appropriate post-hoc test.

Minor points:
5. It is quite puzzling that the tensin knockdown experiments did not include the siRNA-resistant rescue apparently used previously by this lab in a previous paper.

6. In the methods section, with what was the reference mark drawn? Was it drawn on the underside of the dish rather than on the bottom?

7. The wording "…fibrillar adhesions are mechanosensitive" used twice in this paper seems potentially misleading. Focal adhesions are mechanosensitive, e.g., in terms of signaling, but there does not be any evidence presented here for that. Instead, what is shown is that the formation of these adhesions is mechanosensitive.

8. The authors refer to "active" a5b1 integrin, but the SNAKA51 antibody is likely not an integrin activation antibody, but instead one that recognizes a specific conformation of this antibody resulting from binding to fibronectin fibrils. For example, it presumably does not stain at non-fibrillar adhesions when cells are plated on fibronectin coated on a substrate.

9. In line 671, please indicate more clearly what is meant by: "by simply diving the values"

Reviewer 2

Advance summary and potential significance to field

Easier fabrication of gradients than others (but fundamentally the same as Lo et al). They also then find that increasing stiffness promotes the development of fibrillar adhesions that is tensin-dependent.

Comments for the author

This paper from Barber-Pérez et al attempts to create a simple gel system to investigate fibrillar adhesion morphology. I have several concerns, including novelty, below.

1. I am not sure that I believe that the authors have created a "new method" in their hydrogel system. It still relies on the concept of diffusion induced gradients. The only thing different from the Lo paper of 20 years ago is placing the pre-polymerized droplets off center. While I acknowledge that the paper better and more quantitatively characterizes this system, I am not sure how "new" it is.

2a. The non-linear relationship with bead density is troubling. I would like to see the authors first plot modulus versus position to show if the gradient is linear with position and if it is just an issue with bead diffusion. If modulus is non-linear with position AND correlated with bead diffusion, then that is suggestive of the higher concentration polymer retarding bead movement. I would then ask that the authors try a higher Bis-acrylamide, lower acrylamide solution which should have a different viscosity because of lower bulk polymer but which should polymerize to approximately the same modulus (see Wen et al, Nature Materials).
Once that is allowed to diffuse, the gradient may become linear. An alternative would be to allow more mixing time prior to polymerization of the system in Figure 2B. Regardless of how the authors achieve a gradient, I think that to make their system easy for a biologist to use, it should be linear for both.

© 2020. Published by The Company of Biologists under the terms of the Creative Commons Attribution License (https://creativecommons.org/licenses/by/4.0/).

2b. The authors should check non-linearity with position along the orthogonal axis of mixing, i.e. left to right in Figure 1B.

3. It would be helpful if the authors could quantify the change in YAP signaling with position in Figure 2 or use arrows in panel C to highlight the continuous increase in nuclear localization. Overall panels D-F should be shown as a continuum since that is the advantage of the gradient.

4. Why was EDC used when affixing the fibronectin to the substrate? Sulfo-SANPAH alone should be sufficient. Most protocols (see Engler, Gardel, Yu-li Wang, and many others) use EDC and NHS-acrylamide (the latter of which wasn't used) OR Sulfo-SANPAH. Moreover, if the Fibronectin is covalently bound to the substrate, how is it being clustered. Is this cell-generated (EDA) vs. plasma (attached to the substrate - the FN source was not specified) such that it could be clustered? OR is this cells on glass? That is not clear...

5. It is not clear what is new relative to Roca-Cusachs's 2014 Nature Materials paper. The core concepts here are present in that paper, so it is not clear what new concept(s) are elucidated.

Minor
1. HeLa and TIFs are used but no justification is provided for using either or both lines. Especially since both are used to show different things, is that because TIFs don't localize YAP or HeLa's do not have fibrillar adhesions???

Reviewer 3

Advance summary and potential significance to field

This manuscript describes a new method for developing reproducible gradients of stiffness by combining acrylamide hydrogel systems with fluorescent beads establishing a system whereby bead density can be used to calculate gel elasticity. The authors go on to focus on fibrillar adhesions, and their relationship with substrate rigidity, and make some new insight and confirm previous findings. The new methodology will be of interest to the mechanobiology field. The findings related to fibrillar adhesions, whilst limited, are also of interest and perhaps as importantly help to demonstrate the effectiveness of the approach.

Comments for the author

Major comments:
Figure 3: This figure focuses on identifying fibrillar adhesion markers, and does a good job using ratio imaging. It would also be interesting to look at tensin-2, tensin-3, and phospho-FAK to widen the scope and go beyond confirmation of previous findings.
Figures 4 and 5: It's not clear to me if the smaller adhesions are fibrillar adhesions- colocalisation with tensin or fibronectin would be more convincing although it is encouraging that these structures are phospho-paxillin negative. Is there a difference between the stiffness range that promotes fibrillar adhesion growth versus focal adhesions (i.e. vinculin positive)?
Minor comments:
Long sentence lines 202-207 "However…" needs rephrasing to make the meaning clear.

First revision

Author response to reviewers' comments

Reviewer 1 Advance Summary and Potential Significance to Field...
This manuscript describes a creative approach to producing stiffness gradients in very thin polyacrylamide gels that can permit high-resolution imaging. A novel feature is that the predicted

© 2020. Published by The Company of Biologists under the terms of the Creative Commons Attribution License (https://creativecommons.org/licenses/by/4.0/).

stiffness can be determined using embedded micro beads, potentially avoiding the need to perform AFM to calibrate stiffness. The authors use this system to show that (1) a better marker for fibrillar adhesions associated with fibronectin assembly is the SNAKA51 antibody of Clark et al. than the more widely distributed tensin-1, and (2) the formation of fibrillar adhesions is mechanosensitive in the TIF immortalized cell line. Consequently, this study provides a new method for creating stiffness gradients usable for high-magnification microscopy, identifies a good fibrillar adhesion marker, and shows mechanosensitivity of fibrillar adhesion formation.

We thank the reviewer for these encouraging comments.

Reviewer 1 Comments for the Author...
This very well-presented Tools and Resources article provides a cleaver new method for forming very thin polyacrylamide gels with stiffness gradients, confirms and extends information in the previous literature that a particular antibody is a good fibrillar adhesion marker rather than tensin-1, and is the first to show that the formation of fibrillar adhesions is mechanosensitive. Although the latter finding is not surprising given the need for force in organizing fibronectin fibrils, it is novel and useful to establish. The authors of this solid paper should consider the following points in a resubmitted manuscript so that publication can be recommended.

1. A key advance is to provide a new methodology for cell biology labs without ready access to AFM to create stiffness gradients amenable to low working distance microscopy. Several points need clarification:

a) The authors seem to imply that this methodology can be used by other labs using this paper's standard curves to assign Young's moduli without using AFM. If that is the intent, it should be made explicit. If so, this reviewer and readers would prefer to see a little more characterization.
Since 3 experiments were apparently combined for standard curves, it would be useful to see the raw data for each experiment as supplemental data.

That is, the field will want to see the variability in both the data points of bead concentration vs. stiffness, as well as the overall nature of the gradients (e.g., just show the presumably existing images of the fields showing the distribution of beads, probably using stitched-together images), since the simple methods of blending two dots of different polyacrylamide solution is clever but would likely give varying results from experiment to experiment depending on the spacing of the dots and the way in which the coverslip is dropped. In fact, Figure 1 shows a gradient that is diagonally skewed as would be predicted to occur periodically when fusing droplets under a coverslip; the extent of variability would be good to see. Should users discard some gels that do not look even?

We thank the reviewer for this important point. We have now colour coded the individual data points in Fig. 2A-B, such that the relationship between bead density and gel stiffness can be evaluated for all the analysed gradients individually. We also acknowledge that while our protocol is easy and straightforward, the resulting gels and gradients can be somewhat variable. More specifically, the exact location of the gradient in the gel, its slope and orientation relative to the reference mark are all subject to some degree of variation. However, given that the beads provide an internal control such variation does not influence the applicability of the gels for experiments. In contrast, in rare cases, an individual gel might rip, contain bubbles or the gradient itself might be irregular and not entirely smooth. Obviously, such physically damaged gels should be discarded. We have now added images of a whole gradient hydrogel (Fig. S1A), as well as one of the gradients used for creating the standard curves along with respective AFM-measured Young's moduli (Fig. S1C), to give the readers a better idea of what to expect from the method. Moreover, we have added the following to the text (lines 110-115) to encourage users to check their gels before experiments, to discard obviously faulty ones (if any) and e.g. reorient their gels for imaging, if an evenly aligned, top-to-bottom gradient is preferred:

"Due to the nature of the method, individual hydrogels exhibit a degree of variability: for example, the gradient region might not be horizontal across the whole width of the gel (Fig. S1A). In some cases, the gel itself might be damaged or contain air bubbles, or the acrylamide diffusion results in an irregular or 'jagged' gradient; such gels should be discarded. We encourage users to check their gels with a fluorescence microscope before any experiments, and e.g. realign them if a specific orientation of gradient is preferred."

© 2020. Published by The Company of Biologists under the terms of the Creative Commons Attribution License (https://creativecommons.org/licenses/by/4.0/).

With regard to bead gradient, the reviewer is correct in assuming that there is gel-to-gel variability in bead distribution and the mixing of the two polyacrylamide drops; however, we have found that the fundamental bead-stiffness relationship is maintained in different hydrogel preps, and are confident that the fitted results (calculated using data from all 3 repeats) accurately describe this relationship. In only rare occasions, some gradients have been exceptionally irregular (jagged) and these can be discarded.

In addition, the Figure 2C “Beads” image at 4 kPa shows a density of beads at the bottom similar to the uniform distribution at 8 kPa, suggesting variability. These issues may not be a major problem, but it will be important to see the actual data for users to understand what to expect in terms of variability.

We are grateful to the reviewer for pointing this out. In response to this comment and that of another reviewer we have taken a smaller more homogenous bead region, recalculated the stiffness for that specific area and show zoomed-in images to better illustrate YAP/TAZ localisation.

b) This reviewer is quite surprised at the tight fitting of the AFM data in supplemental figure 1 to the predicted curve at the very low and high stiffness zones, especially considering the comment on lines 143-144 stating that “significant changes in stiffness were not accompanied by changes in bead density.”. But if values in these regions are reliable, this method could be used for a wide range of stiffnesses, but is that not the case?

This is a valuable point that calls for some clarification. We would like to draw the reviewer’s attention to the comparatively steep slope of the standard curve in Fig. 2B near the minimum and especially maximum substrate stiffness. As such, the relative error in stiffness (i.e. distance of the confidence interval from the best fit specifically along the y-axis) is in fact larger than the first impression might suggest. This is especially true for the softest part of the gradient, where the 95% CI for the predicted 2.5 kPa stiffness spans from ~0 to ~10 kPa. In other words, the numbers *are* reliable but come with a reasonable degree of uncertainty. The quantity of individual measurements/data points also contributes to a tighter, more reliable fit and better prediction of the bead-stiffness relationship. The comment on the lines 143-144 was indeed misleading, and we have now modified it (lines 140-146 in the new version of the manuscript) as indicated in our reply to the reviewer’s next concern.

As to the reviewer’s question on whether this method could be used for a wide range of stiffnesses, this is exactly what we had hoped to accomplish and the reason why we chose two very different stiffness gradients. However, we demonstrated that the relationship between bead density and stiffness is different in our narrow range and wide range stiffness gradient gels. This is something that needs to be taken into consideration if testing out other gradients than those we have investigated here. The strength of our study lies in bringing this important point to the attention of readers and in providing two tested stiffness ranges that have been validated and could be used by others. The weakness is that we cannot speculate at what range the linearity between bead density and gel stiffness would change and, therefore, we recommend that any new stiffness gradients be validated with AFM before use.

c) Most importantly, it is not clear to this reviewer how there can be such non- linear relationships between bead concentration and AFM-measured stiffness, since the bead concentration should be a simple proxy for polyacrylamide concentration. This problem ideally needs some explanation beyond the very unclear comment about large differences in stiffness of the starting gels (lines 144-145) , since such non-linearity seems to imply that it may not be safe to use the authors’ standard curves for other than narrow stiffness ranges.

Here, too, the reviewer raises a very important point. One of our key aims was that the method could be used for a wide variety of stiffnesses, which is why we chose to work with two very different stiffness gradients. However, as pointed out by the reviewer, the relationship between bead density and stiffness was quite different between our narrow range and wide range gradient gels. The mechanistic explanation for this is not clear, although one could hypothesize that the faster polymerization of the more concentrated acrylamide (Buxboim et al., 2010; PMID: 20454525) could

© 2020. Published by The Company of Biologists under the terms of the Creative Commons Attribution License (https://creativecommons.org/licenses/by/4.0/).

affect its diffusion kinetics, while the beads, essentially acting as a permeant in the polymer at this stage, would not be restricted by covalent interactions to a similar degree, and could diffuse following partially different kinetics. We think that the observation is interesting and important to keep in mind when working with hydrogel-based techniques such as ours; as we cannot speculate at what range the linearity between bead density and hydrogel stiffness would change, we recommend that any new stiffness gradients be validated by AFM before use. However, we feel that finding out the actual physical explanation for the phenomenon would be laborious and beyond the scope of this work.

We want to emphasize that regardless of its non-linearity, the standard curve for wide range stiffness gradients can be used to predict hydrogel stiffness at a given bead density, reproducibly and with a sufficient degree of precision, across the full length of the gradient (Fig. 2B). Because of this, we do feel that our standard curves, as well as the respective stiffness gradient hydrogels, are safe to use for both narrow and wide stiffness ranges as long as the usual statistical considerations are taken into account. As to why no one else has reported this nonlinearity before, it might be because, to the best of our knowledge, diffusion-based stiffness gradients above 30 kPa (30 kPa in Lo et al.,) have not been studied before. In order to clarify all of the above points, we have modified/added the following passages:

Lines 140 - 146: *"In comparison, we found that the wide-range stiffness (2 – 60 kPa) correlation curve exhibited a more complex relationship between bead density and gel stiffness, best modelled as a logit curve (Fig. 2B). At the two extremes of the gradient, relatively small differences in bead density were accompanied by larger changes in stiffness. Conversely, at intermediate bead densities the relationship was more linear and beads were a better overall predictor of substrate stiffness."*

Lines 280 - 287 *"It is unclear whether this non-linear relationship results from partially different diffusion kinetics between acrylamide and the fluorescent marker beads, a phenomenon that is then exacerbated by the increased acrylamide concentration in the wide range gradients. To our knowledge, no diffusion-based polyacrylamide stiffness gradients with elastic moduli reaching up to 60 kPa have been reported before, for example, when fluorescein was used as a means to measure hydrogel stiffness (range of 0.1 – 10 kPa; (Koser et al., 2016)). This observation is therefore important to keep in mind for any future modifications of the technique."*

2. For this methodology to be useful to other labs, the three custom scripts for the processing approaches will need to be readily available. Will they be available from a public database with instructions for use? Without such methods and a reliable pair of standard curves, this methodology will not be sufficiently useful to the field.

We have now provided access to all the relevant scripts needed to process images through GitHub at the following address: https://github.com/Ivaska-Lab-UTU/StiffnessGradientHydrogels

3. How thick are the polyacrylamide gels (beyond just less than 100 um)? That the thickness can matter is shown by the Buxboim-Discher paper PMID: 20454525, in which proximity to the substrate can affect what cells can sense. Some reassurance that this issue will never be a problem would be helpful.

We fully agree that the thickness of the gel is of paramount importance and can determine whether the cell is in fact sensing the rigid glass surface under the gel rather than the gel itself. We confirm (new Figure S1B) that our hydrogels exhibit a thickness above 100 µm and thus proximity to the glass coverslip is not a confounding issue in our experiments. Our initial estimate of less than 100 µm was based on the volume pipetted onto the coverslip and had not taken into account hydrogel swelling. In the new analysis, we measured gel thickness at 12 regions of interest within a bead gradient from two different hydrogels and these measurements show that the gel thickness is in a range between 100 - 150 µm. We have now added the following passage (lines 117 - 122) to reflect this:

"Cells cultured on very thin (<20 micron) polyacrylamide hydrogels may be able to "feel" the underlying rigid glass or plastic, leading to confounded mechanosensing on such substrates (Buxboim et al, 2010). We measured the thickness of our stiffness gradient hydrogels along the length of the gradient and found it to be in the range of 100-150 µm, thick enough to prevent cells from being influenced by the glass but still amenable to high-resolution imaging (Fig. S1B)."

© 2020. Published by The Company of Biologists under the terms of the Creative Commons Attribution License (https://creativecommons.org/licenses/by/4.0/).

4. The use of statistics for Figure 3D was likely incorrect because there were three sets of data compared rather than a single pair. The authors should either use the Bonferroni correction for t-tests or ANOVA with an appropriate post-hoc test.

We apologise about the mistake in reporting. The test used was actually ANOVA followed by Tukey's honestly significant difference (HSD).

Minor points: 5. It is quite puzzling that the tensin knockdown experiments did not include the siRNA-resistant rescue apparently used previously by this lab in a previous paper.

This would have been an ideal experiment but very difficult to achieve with TIFFs in this setting. This is because TIFFs have a very poor plasmid transfection efficiency. In the previous paper, rightly pointed out by the reviewer, we were able to overcome the low number of GFP-tensin transfected cells by: i) seeding a large number of cells in 10 cm plates and ii) performing integrin activity (our biological readout) assays using flow cytometry where cells are selected for GFP expression, gated, and analysed for integrin activity. In this manuscript, we are relying on immunofluorescence, and the number of cells that can be plated on the hydrogels is by no means comparable to what can be achieved in normal culture plates. Therefore, the chances of finding a sufficient number of GFP-tensin-positive TIFFs evenly distributed along the gradient for analysis is very low and we did not perform the experiment.

6. In the methods section, with what was the reference mark drawn? Was it drawn on the underside of the dish rather than on the bottom?

The reference mark was drawn on the underside of the dish with a permanent marker. We have now made this clear in the text.

7. The wording "…fibrillar adhesions are mechanosensitive" used twice in this paper seems potentially misleading. Focal adhesions are mechanosensitive, e.g., in terms of signaling, but there does not be any evidence presented here for that. Instead, what is shown is that the formation of these adhesions is mechanosensitive.

We thank the reviewer for this comment. We do not wish to mislead the reader and have thus modified the text accordingly.

8. The authors refer to "active" a5b1 integrin, but the SNAKA51 antibody is likely not an integrin activation antibody, but instead one that recognizes a specific conformation of this antibody resulting from binding to fibronectin fibrils. For example, it presumably does not stain at non-fibrillar adhesions when cells are plated on fibronectin coated on a substrate.

We had used the word "active" to describe the conformation of the integrin that is detected by this antibody (a primed integrin capable of binding ligand). To the best of our knowledge, SNAKA51 is not restricted to integrins bound to fibronectin fibrils; it has been used in the literature to detect active α5β1 in endosomes, in immunoprecipitations from cell lysates and in flow cytometry in suspended cells, It is also important to note that all the experiments with the SNAKA51 antibody are done by staining this integrin conformer in fixed cells. Thus, in this context, the antibody would no longer be able to activate integrins as shown in the original paper describing this antibody but would still recognise the active/open conformation (Clark et al., 2005). However, we realise now, as pointed out by the reviewer, that this may be misconstrued by readers. Therefore, we have tried to make the distinction clear in the text on lines 176-177.

"We confirmed that tensin-1 and active α5β1-integrin (labelled with the SNAKA51 antibody, recognising the primed conformation of the receptor in these fixed cells), previously reported to be enriched at fibrillar adhesions, demonstrate equal abundance in centrally located adhesions (Fig.3A)"

9. In line 671, please indicate more clearly what is meant by: "by simply diving the values"

© 2020. Published by The Company of Biologists under the terms of the Creative Commons Attribution License (https://creativecommons.org/licenses/by/4.0/).

We have now edited the text for clarity to "*by dividing, pixel by pixel, the values of the first channel by the second channel*".

Reviewer 2 Advance Summary and Potential Significance to Field...

Easier fabrication of gradients than others (but fundamentally the same as Lo et al). They also then find that increasing stiffness promotes the development of fibrillar adhesions that is tensin-dependent.

Reviewer 2 Comments for the Author...

This paper from Barber-Pérez et al attempts to create a simple gel system to investigate fibrillar adhesion morphology. I have several concerns, including novelty, below.

1. I am not sure that I believe that the authors have created a "new method" in their hydrogel system. It still relies on the concept of diffusion induced gradients. The only thing different from the Lo paper of 20 years ago is placing the pre-polymerized droplets off center. While I acknowledge that the paper better and more quantitatively characterizes this system, I am not sure how "new" it is.

We are grateful for this comment. The reviewer is correct in indicating that the Lo et al paper also used fluorescent beads to define gel rigidity areas. However, they used the beads as a way to distinguish stiff from soft extremes not as a means to calculate absolute stiffness. We would like to point out that we are clearly indicating in the manuscript that our method is based on the Lo et al paper. However, our method is more detailed, and shows that by imaging beads, we can mathematically relate bead density to hydrogel stiffness. In contrast, in Lo et al, AFM is still required to measure the stiffness. The novelty of our work comes from the possibility to precisely calculate the Young's modulus at all locations of a gradient without the need for complex instruments such as an AFM.

We have changed the word "new" to "modified" on lines 99-101 to alleviate further the reviewer's concerns. *"Towards this goal, we took elements from other approaches (Koser et al., 2016; Lo et al., 2000), and developed a modified method to generate stiffness gradient hydrogels."*

2a. The non-linear relationship with bead density is troubling. I would like to see the authors first plot modulus versus position to show if the gradient is linear with position and if it is just an issue with bead diffusion. If modulus is non-linear with position AND correlated with bead diffusion, then that is suggestive of the higher concentration polymer retarding bead movement. I would then ask that the authors try a higher Bis-acrylamide, lower acrylamide solution which should have a different viscosity because of lower bulk polymer but which should polymerize to approximately the same modulus (see Wen et al, Nature Materials). Once that is allowed to diffuse, the gradient may become linear. An alternative would be to allow more mixing time prior to polymerization of the system in Figure 2B. Regardless of how the authors achieve a gradient, I think that to make their system easy for a biologist to use, it should be linear for both.

2b. The authors should check non-linearity with position along the orthogonal axis of mixing, i.e. left to right in Figure 1B.

We thank the reviewer for raising this important point. The diffusion and gradual polymerization of acrylamide solutions should, in all likelihood, both contribute to the layout of the resulting polyacrylamide (PAA) and the profile of the stiffness gradient. Factor in the fluorescent microbeads that are not associating covalently with the PAA matrix, and it is plausible that the polymer and its "permeant" have at least partially distinct diffusion kinetics. This phenomenon may be exacerbated by the higher concentration of acrylamide interfering with bead diffusion directly, as suggested by the reviewer, and/or simply the faster polymerization of more concentrated acrylamide solution (Buxboim et al 2010; PMID: 20454525) affecting monomer diffusion.

To alleviate some of the reviewer's concerns about using our gradients for biological experiments, we have plotted AFM-derived substrate stiffness as a function of location along the gradient in the figure below (for Reviewer only): the relationship is mostly linear and only slightly sigmoid for both narrow and wide range stiffness gradients. This indicates that our gradients should be amenable for

© 2020. Published by The Company of Biologists under the terms of the Creative Commons Attribution License (https://creativecommons.org/licenses/by/4.0/).

any experiment, excluding those where absolute control over the slope of the gradient is needed. On the other hand, the shape of the gradient (i.e. its orientation across the full width of the hydrogel) can indeed vary between gels. We have now added tile scan images of a complete gradient hydrogel (Fig. S1A) and one of the narrow range gradients used for optimization, along with its respective AFM measurements (Fig. S1C), to convey better to the reader what to expect from the method. The users are encouraged to check their hydrogels before starting experiments, and realign them for imaging if a specific orientation of gradient is needed.

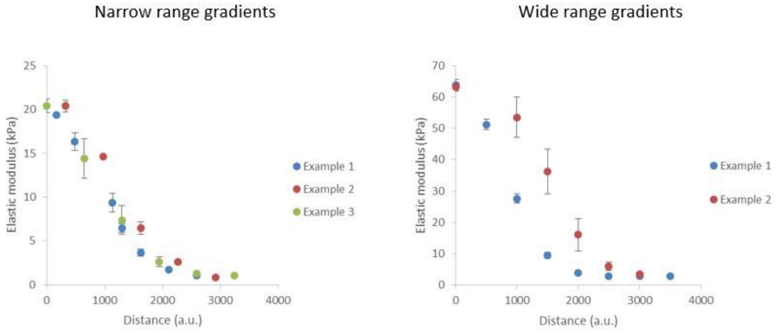

Figure for Reviewer only: AFM-derived substrate stiffness as a function of location along the gradient

One of our key aims was that the method could be used for a wide variety of stiffnesses, which is why we chose to work with two very different stiffness gradients. However, as pointed out by the reviewer, the relationship between bead density and stiffness was quite different between our narrow range and wide range gradient gels. While the mechanistic reason for this is not clear, we think that the observation is interesting and important to keep in mind when working with hydrogel-based techniques like ours; since we cannot state the range where linearity between bead density and hydrogel stiffness begins to change, we recommend that any new stiffness gradients be validated by AFM before use. However, we also feel that finding out the actual physical explanation for the phenomenon would be laborious and beyond the scope of this work.
We want to emphasize that regardless of its non-linearity, the standard curve for wide range stiffness gradients can be used to predict hydrogel stiffness at a given bead density, reproducibly and with a sufficient degree of precision, across the full length of the gradient (Fig. 2B). Because of this, we do feel that our standard curves, as well as the respective stiffness gradient hydrogels, are safe to use for both narrow and wide stiffness ranges as long as the usual statistical considerations are taken into account. In order to clarify all of the above points, we have modified the following passages:

Lines 140 -146: *"In comparison, we found that the wide-range stiffness (2 – 60 kPa) correlation curve exhibited a more complex relationship between bead density and gel stiffness, best modelled as a logit curve (Fig. 2B). At the two extremes of the gradient, relatively small differences in bead density were accompanied by larger changes in stiffness. Conversely, at intermediate bead densities the relationship was more linear and beads were a better overall predictor of substrate stiffness."*

Lines 280 - 287 *"It is unclear whether this non-linear relationship results from partially different diffusion kinetics between acrylamide and the fluorescent marker beads, a phenomenon that is then exacerbated by the increased acrylamide concentration in the wide range gradients. To our knowledge,*

no diffusion-based polyacrylamide stiffness gradients with elastic moduli reaching up to 60 kPa have been reported before, for example, when fluorescein was used as a means to measure hydrogel stiffness (range of 0.1 – 10 kPa; (Koser et al., 2016)). This observation is therefore important to keep in mind for any future modifications of the technique."

The reviewer also made a compelling suggestion of using less acrylamide and more crosslinker (bis-acrylamide) to reduce viscosity of the pre-polymer solution. This could possibly alleviate the issue of non-linear gel-stiffness relationship seen in the wide range stiffness gradients. The approach seems plausible but would, at this stage, mean repetition of all experiments, revalidation and reanalysis,

© 2020. Published by The Company of Biologists under the terms of the Creative Commons Attribution License (https://creativecommons.org/licenses/by/4.0/).

which was not feasible within the timeframe of revisions.

3. It would be helpful if the authors could quantify the change in YAP signaling with position in Figure 2 or use arrows in panel C to highlight the continuous increase in nuclear localization. Overall panels D-F should be shown as a continuum since that is the advantage of the gradient.

These were preliminary validation experiments performed to test our system and we regret that we did not image enough gels/cells to show confidently YAP/TAZ localisation in response to stiffness as a continuum. The quant in 2D-2F was possible because we pooled the data from below 1 kPa (soft) and from above 8 kPa (stiff) to show the changes in cell YAP/TAZ nuclear localisation and in cell morphology. As stiffness-mediated regulation of YAP/TAZ is well documented in the literature it was not the focus of our study, it was included here merely as a proof-of-concept and we did not go any further. In response to this reviewer and another reviewer, we have now recalculated the stiffness of the images over a smaller area, showing a magnified region to better illustrate YAP/TAZ localisation in Figure 2C.

We would like to point out that the focus of the study, the formation of fibrillar adhesion formation, is carefully analysed over a continuum.

2. Why was EDC used when affixing the fibronectin to the substrate? Sulfo-SANPAH alone should be sufficient. Most protocols (see Engler, Gardel, Yu-li Wang, and many others) use EDC and NHS-acrylamide (the latter of which wasn't used) OR Sulfo-SANPAH.

Regarding the combination of Sulfo-SANPAH and EDC (N, N-(3-dimethylaminopropyl)-N-ethyl carbodiimide), this is a novel protocol that our lab has optimized (first used in Lerche et al 2019, iScience and then in Stubb et al, 2020, Nano letters). While other protocols normally combine EDC and NHS for gel functionalization, in our hands the combination of EDC+sulfo-SANPAH was more optimal than sulfo-SANPAH alone or EDC+NHS. We found that using this protocol we could saturate hydrogels using a reasonable amount of ECM ligand.

3. Moreover, if the Fibronectin is covalently bound to the substrate, how is it being clustered. Is this cell-generated (EDA) vs. plasma (attached to the substrate - the FN source was not specified) such that it could be clustered? OR is this cells on glass? That is not clear...

Any coating of glass coverslips/gels was performed with bovine fibronectin (cells were also plated in full cell culture medium containing serum). However, in the long-term, and without any inhibition of de novo protein synthesis, cells will deposit their own fibronectin matrix as is the case in our experiments.

4. It is not clear what is new relative to Roca-Cusachs's 2014 Nature Materials paper. The core concepts here are present in that paper, so it is not clear what new concept(s) are elucidated.

As far as we are aware, this paper focuses on investigating traction-forces, bond lifetimes and focal adhesion maturation as a response to rigidity. They also focus on the contributions of two distinct integrin types, α5β1 and αvβ6, in dictating the traction optimum of mammary myoepithelial cells. We agree that this paper and subsequent follow-up papers from them and others have established clearly the concept of focal adhesion maturation in response to increasing matrix rigidity. However, to the best of our knowledge the concept that the formation of fibrillar adhesions (these are distinct from focal adhesions in their composition, life-time, force-transmission and the biological function and are predominantly only generated in mesenchymal cells like fibroblasts) is mechanosensitive is new.

5. Minor 1. HeLa and TIFs are used but no justification is provided for using either or both lines. Especially since both are used to show different things, is that because TIFs don't localize YAP or HeLa's do not have fibrillar adhesions???

We were using HeLa cells during the initial testing and optimisation of the hydrogels. We used YAP/TAZ nuclear localisation as a preliminary proof-of-concept readout because it is a well-reported phenomenon in response to changes in stiffness. When our protocol for producing gels was working,

© 2020. Published by The Company of Biologists under the terms of the Creative Commons Attribution License (https://creativecommons.org/licenses/by/4.0/).

we chose to investigate the formation of fibrillar adhesions, as a continuation from one of our previous papers (Georgiadou et al., 2017), and therefore switched our cell model to fibroblasts, which contain prominent fibrillar adhesions and are the main fibronectin-depositing cells within tissues.

Of note, the regulation of YAP/TAZ activity is an important pathway in fibroblasts, especially in fibrotic disease. However, this pathway was not the focus of this paper and we did not repeat the YAP/TAZ localisation assays in TIFFs.

The fact that we have used two very different cell lines to investigate biologically distinct processes: nuclear localisation of mechanosensitive transcription factors and formation of a specific matrix-depositing adhesion types does however demonstrate the broad application of our hydrogels.

Reviewer 3 Advance Summary and Potential Significance to Field...

This manuscript describes a new method for developing reproducible gradients of stiffness by combining acrylamide hydrogel systems with fluorescent beads, establishing a system whereby bead density can be used to calculate gel elasticity. The authors go on to focus on fibrillar adhesions, and their relationship with substrate rigidity, and make some new insight and confirm previous findings. The new methodology will be of interest to the mechanobiology field. The findings related to fibrillar adhesions, whilst limited, are also of interest and perhaps as importantly help to demonstrate the effectiveness of the approach.

Reviewer 3 Comments for the Author...

Major comments: Figure 3: This figure focuses on identifying fibrillar adhesion markers, and does a good job using ratio imaging. It would also be interesting to look at tensin-2, tensin-3, and phospho-FAK to widen the scope and go beyond confirmation of previous findings.

Thank you for this great suggestions. We have now made additional staining for the following: Tensin3+Vinculin, pFAK+Vinculin and pFAK+SNAKA51. The antibodies against Tensin3 and pFAK are both raised in rabbit, therefore we could not directly compare the localisation of these with fibronectin. We added the following information to the manuscript:

Lines 193-198: *"However, active a5β1-integrin, which demonstrated a strong overlap with fibronectin in centrally located adhesions and is absent from peripheral adhesions, also showed limited colocalization with phospho-paxillin and phospho-FAK (Fig. 3C, D and Fig S3C). Thus, in line with fibrillar adhesions being viewed as phosphotyrosine poor structures within the cell (Zamir et al., 2000), active a5β1-integrin appears to be a more appropriate fibrillar adhesion marker."*

Lines 184-186: *"Tensin3, the other tensin isoform reported to be enriched in fibrillar adhesions also overlapped with vinculin in peripheral adhesions (Fig. S2C)."*

With regard to Tensin2, to the best of our knowledge there are no good immunofluorescence antibodies for this protein. In addition, as tensin1 and tensin3 are reported to be in fibrillar adhesions, we focused on these two family members.

Figures 4 and 5: It's not clear to me if the smaller adhesions are fibrillar adhesions- colocalisation with tensin or fibronectin would be more convincing although it is encouraging that these structures are phospho-paxillin negative.

There is no clear definition or rather defining marker of fibrillar adhesions. In Figure 3, we made careful comparison between proteins reported in the literature to either localise to or to be absent from fibrillar adhesions. Based on these assessments - highest correlation between SNAKA51 and fibronectin localisation, and absence of pPaxillin in SNAKA51 adhesions - we chose SNAKA51 as the marker to be used in Figures 4 and 5. We agree that including co-staining of a second marker, fibronectin, may have further strengthened the accuracy of fibrillar adhesion definition. However, we felt that this was not feasible or expected to alter significantly the outcome of the study. Using two antibodies and their co-localisation analysis on top of bead quantification and adhesion length scoring would have significantly complicated the analysis pipeline. As we indicate, tensin 1 appears in focal adhesions as well as in fibrillar adhesions to a greater degree than SNAKA51 based on

© 2020. Published by The Company of Biologists under the terms of the Creative Commons Attribution License (https://creativecommons.org/licenses/by/4.0/).

ratiometric analysis with focal adhesion markers and thus we avoided using tensin as a marker.

Despite our finding, we have deliberately tried to call the smaller adhesions, mentioned by the reviewer, “active α5β1 integrin adhesions” instead of fibrillar adhesions to avoid misleading the reader. We apologize if we had missed a few of these cases in the text, which we have now tried to rectify.

Is there a difference between the stiffness range that promotes fibrillar adhesion growth versus focal adhesions (i.e. vinculin positive)?

The idea that the growth of fibrillar adhesions may be favoured over focal adhesions after a certain stiffness threshold is extremely interesting and could be one mechanism contributing to increased fibronectin secretion in fibrotic diseases. However, we did not investigate this in any quantitative manner and believe further experiments and controls are needed to give the reviewer a definitive response. At this point, we believe investigations into fibrillar versus focal adhesion length are beyond the scope of this manuscript but a subject we wish to study in the future using our hydrogels.

Minor comments: Long sentence lines 202-207 “However…” needs rephrasing to make the meaning clear.

The sentence has been divided into two shorter sentences (now lines 193-198).

Second decision letter

MS ID#: JOCES/2019/242909

MS TITLE: Mechano-responsiveness of fibrillar adhesions on stiffness-gradient gels

AUTHORS: Nuria Barber-Perez, Maria Georgiadou, Camilo Guzman, Aleksi Isomursu, Hellyeh Hamidi, and Johanna Ivaska
ARTICLE TYPE: Tools and Resources

I hope you and your family as well as everyone in your lab are safe and well at this difficult time. I had indicated that I would send your paper back to the reviewers. However, going through your revisions and thorough responses to the reviewers questions I feel that there is no need to send the paper back out. So I am happy to tell you that your manuscript has been accepted for publication in Journal of Cell Science, pending standard ethics checks.

© 2020. Published by The Company of Biologists under the terms of the Creative Commons Attribution License (https://creativecommons.org/licenses/by/4.0/).
